# Peritumoral B cells drive proangiogenic responses in HMGB1-enriched esophageal squamous cell carcinoma

**DOI:** 10.1007/s10456-021-09819-0

**Published:** 2021-10-06

**Authors:** Ngar Woon Kam, Ka Chun Wu, Wei Dai, Ying Wang, Leo Yik Chun Yan, Reshma Shakya, Rajiv Khanna, Yanru Qin, Simon Law, Anthony Wing Ip Lo, Victor Ho Fun Lee, Xin-Yuan Guan, Dora Lai-Wan Kwong

**Affiliations:** 1 Department of Clinical Oncology, Li Ka Shing Faculty of Medicine, The University of Hong Kong, Queen Mary Hospital, 1/F, Professorial Block, 102 Pokfulam Road, Pok Fu Lam, Hong Kong; 2 Laboratory for Synthetic Chemistry and Chemical Biology Limited, Hong Kong Science and Technology Parks, Sha Tin, New Territories Hong Kong; 3 Department of Medicine and Therapeutics, Faculty of Medicine, Li Ka Shing Institute of Health Sciences, The Chinese University of Hong Kong, Pok Fu Lam, Hong Kong; 4 QIMR Centre for Immunotherapy and Vaccine Development and Department of Immunology, QIMR Berghofer Medical Research Institute, Brisbane, QLD 4029 Australia; 5 Department of Clinical Oncology, The First Affiliated Hospital, Zhengzhou University, Zhengzhou, China; 6 Department of Surgery, Li Ka Shing Faculty of Medicine, The University of Hong Kong, Pok Fu Lam, Hong Kong; 7 Department of Anatomical Pathology, Queen Mary Hospital, Pok Fu Lam, Hong Kong

**Keywords:** B cells, High-mobility group box 1, Angiogenesis, Esophageal squamous cell carcinoma

## Abstract

Several B-cell subsets with distinct functions and polarized cytokine profiles that extend beyond antibody production have been reported in different cancers. Here we have demonstrated that proliferating B cells were predominantly found in the peritumoral region of esophageal squamous cell carcinoma (ESCC). These B cells were enriched in tumor nests with high expression of high-mobility group box 1 (HMGB1). High densities of peritumoral proliferating B cells and concomitantly high intratumoral HMGB1 expression showed improved prognostic significance, surpassing prognostic stratification of ESCC patients based on HMGB1 positivity alone. This striking association led us to set up models to test whether cancer-derived HMGB1 could shape tumor microenvironment via modulation on B cells. Overexpression of HMGB1 in ESCC cell lines (KYSE510 and EC18) enhanced proliferation and migration of B cells. Transcriptomic analysis showed that migratory B cells exhibited high enrichment of proangiogenic genes. VEGF expression in proliferating B cells was induced upon co-culture of HMGB1-overexpressing tumor cells and B cells. Secretome array profiling of conditioned media (CM) from the co-culture revealed rich expression of proangiogenic proteins. Consequently, incubation of human umbilical vein endothelial cells with CM promoted angiogenesis in tube formation and migration assays. HMGB1 inhibitor, glycyrrhizin, abolishes all the observed proangiogenic phenotypes. Finally, co-injection of B cells and CM with HMGB1-overexpressing tumor cells, but not with glycyrrhizin, significantly enhanced tumor growth associated with increased microvascular density in ESCC xenograft mice model. Our results indicate that cancer-derived HMGB1 elevates angiogenesis in ESCC by shifting the balance toward proangiogenic signals in proliferating B cells.

**Supplementary Information:**

The online version contains supplementary material available at 10.1007/s10456-021-09819-0.

## Introduction

Esophageal cancer is one of the most common cancers worldwide, with higher incidence rates in Eastern Asia and in Eastern and Southern Africa, in which esophageal squamous cell carcinoma (ESCC) accounts for the predominant histological type [1]. Despite improvements in its detection and treatment, the prognosis in patients with ESCC remains poor, with an overall 5-year survival rate of 15–34% [2, 3]. Within the complex tumor microenvironment (TME), intercellular communication between malignant and other cells of the host is prerequisite during tumorigenesis to facilitate cancer growth [4]. In the era of personalized cancer medicine and innovative immunotherapeutic strategies, profound knowledge of the dynamics of CD8^+^ T cells has regained considerable interest. However, tumor vasculature and stromal components within the TME may pose a barrier against intratumoral trafficking of CD8^+^ T cells [5, 6]; therefore, T cell therapies are modestly efficacious and patients often develop resistance. For this reason, additional therapeutic interventions are required for non-T cell-inflamed tumors to appropriately remodel the TME to render these tumors more sensitive to cancer treatments [7].

We have recently shown an increased infiltration of type 2 macrophages in tumor stroma of FOXO1-positive ESCC tissue [8], while others have reported high accumulation of CD20^+^ B cells in the tumor nest of ESCC [9] and that abundant intratumoral CD20^+^ B cells and CD8^+^ T cells are associated with better outcomes in patients with oropharyngeal squamous cell carcinoma (OPSCC) [10]. In fact, a wide range of cancers become infiltrated with B cells, and separate studies have reported that tumor-promoting and tumor-inhibitory functions of B cells play important roles in tumor progression [11–16]. Recent reports document that B cells are also important for tumor angiogenesis [17]. Therefore, the dynamic TME necessitates the study of the interactions of tumor, immune cells, and vascular endothelial cells (ECs).

Tumor angiogenesis is the formation of new networks of blood vessels induced by tumor-secreted factors and is implicated in tumor progression [18–20]. Growth of solid tumors is highly dependent on newly formed blood vessels and this tumorigenic neovascularization requires several steps, including activation of vascular ECs and degradation of the basal membrane and extracellular matrix [21]. Hypoxia is related to oxidative stress and enhancing cancer-associated angiogenesis [22]. It is also recognized as a major obstacle to the success of cancer immunotherapy. A protein frequently upregulated in hypoxic conditions is high-mobility group box 1 (HMGB1) [23, 24]. HMGB1 is a conserved evolutionarily DNA-binding nuclear protein that has been represented as a damage-associated molecular pattern (DAMP) protein involved in several disease states, including sepsis [25], arthritis [26], and cancer [27]. Tumor cells can release HMGB1 into the local microenvironment, where HMGB1 interacts with several receptors, such as toll-like receptor 2 (TLR-2), TLR-4, TLR-9, and the receptor for advanced glycation end products (RAGE), which can lead to tumor cell survival, proliferation, and angiogenesis [3, 28, 29]. Overexpression of HMGB1 in tumor tissue or elevation of HMGB1 levels in the serum of cancer patients is associated with poor prognosis in various malignancies [30–34]. Regarding ESCC, it has been reported that HMGB1 was overexpressed in the tumor and plasma of ESCC patients, and high levels of HMGB1 in ESCC tissues were associated with tumor progression, poor prognosis [35, 36], and development of radioresistance [37, 38]. Moreover, HMGB1 mediates tumor immune escape by promoting the proliferation and differentiation of myeloid-derived suppressor cells [39], inducing regulatory T cells [40] or B cells [41]. However, whether HMGB1 expressed on tumor cells can induce pro- or antitumorigenic B-cell production in ESCC is, however, totally unknown.

Based on these data, we designed a study aimed at identifying the role of cancer-derived HMGB1 in the immune contexture of the TME, with particular focus on B cells, which could potentiate the tumorigenicity of ESCC through angiogenesis.

## Results

### Increased frequencies of proliferating B cells in the peritumoral region of ESCC compared to those present in the non-tumoral adjacent tissue

**Fig. 1:**
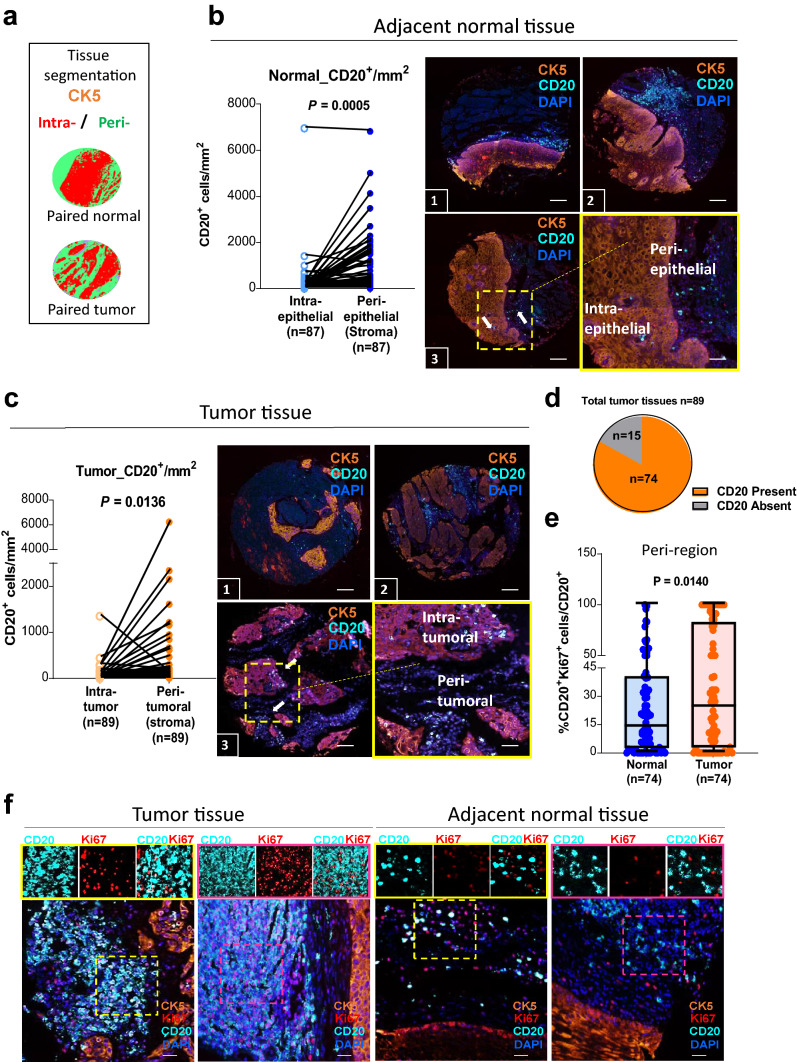
High proportions of proliferating B cells are found in the peritumoral region of ESCC tissues. **a** Representative image of machine learning-based image processing for tissue category classification (intra-, red; peri- green). **b**,** c** The density of CD20^+^ B cells as counts/mm^2^ in 89 paired normal (**b**, blue) and tumor (**c**, orange) tissues of ESCC. TMA stained with tumor marker (cytokeratin-5, CK5, orange), B-cell marker (CD20, turquoise), and nuclei (DAPI, blue). Scale bar: 100 µm. Three individual representative TMA cores of tumor and normal tissues are shown. Three-color overlay (top two and bottom left) and enlarged insets (border) of a representative sampling of the simultaneously acquired markers. Density of CD20^+^ B cells/mm^2^ was higher in peri-region than intra-region in both tumor (light orange vs. dark orange) and normal (light blue vs. dark blue) tissues. The sample size in normal was 87 due to deficit data on CK5 of two cores in normal tissues. **d** 15 out of 89 patients did not have CD20^+^ cells and were removed from the subsequent analysis. **e** Proliferating B cells (%CD20^+^Ki67^+^ cells/total CD20^+^ cells) were higher in the peritumoral region of esophageal cancer than in normal (*n* = 74). **(f)** Representative images of two tumor and two normal tissues are shown. Tissues stained for Ki67 (red), CD20 (turquoise) in peri-region (CK5^−^, orange) and counterstained with DAPI. In ESCC tumor tissues, higher amount of peritumoral B cells co-expressed with Ki67 (proliferating B cells), while in the peri-region of normal tissue, most of the B cells are not proliferative. Magnified insets with representative region of individual markers (or select combinations of markers) are shown on the side. Scale bar: 50 µm

Within tumor tissue, the spatial characteristics of immune cells are one of the important determinants of immune cell function. We used a tissue microarray (TMA) containing 89 paired tumor/adjacent normal formalin-fixed paraffin-embedded (FFPE) ESCC tissues to evaluate the density and spatial distribution of CD20^+^ B cells by multiplex immunohistochemistry (mIHC). Using the tissue segmentation function, the intra-(tumor or epithelial) region was defined by cytokeratin-5 (CK5^+^ epithelial cells) and the peri-(tumor or epithelial) region in stroma comprised CK5^−^ non-epithelial cells (Fig. 1a). We observed a higher density of B cells in stroma than in intra-epithelial locations in normal tissues (90%; 79/87, two cases were excluded due to a deficit of data on cytokeratin-5; Fig. 1b). Similar findings were observed in tumors where B cells were frequently found in peritumoral location instead of intratumoral region (88%; 79/89; Fig. 1c). For 15 of the 89 study patients, CD20 immunostaining was not evident in the tumor cores (Fig. 1d). For the 74 cases with peritumoral B-cell infiltration, we examined the proliferation marker Ki67 in B cells (hereafter referred to as proliferating B cells). The proportion of proliferating B cells in peritumoral compartment was significantly higher than that in corresponding normal tissues (median: 24.16 ± 4.50 vs. 13.59 ± 3.18, respectively, Fig. 1e–f and Supplementary Fig. 1a).

To further assess the clinical significance of non-T cell (CD3^+^) abundance, we evaluated the density of tumor-infiltrating lymphocytes (TILs) derived from a cell suspension (via flow cytometry) in four paired resected tumors and adjacent cancer-free normal tissues. The analysis showed that the percentages of total CD3^–^ cells were significantly higher than CD3^+^ cells, (2.71-fold for normal; p = 0.0267 vs. 3.68-fold for tumor; p = 0.0185), but no significant differences were found in the frequencies of total CD3^+^ and CD3^–^ cells between tumor and normal tissue (median: 18.48 ± 12.28 vs. 25.13 ± 7.51 and 67.93 ± 12.89 vs. 71.00 ± 6.99, respectively; Supplementary Fig. 1b). This finding was confirmed by RNA sequencing (RNA-seq) of three paired ESCC tumor and normal tissues (Supplementary Fig. 1c), indicating that CD3^–^ immune cells (non-T cells) are enriched in ESCC irrespective of whether tissues are malignant or benign. We previously reported that 22 types of TILs were identified in The Cancer Genome Atlas (TCGA) database of ESCC patients [8]. Here, we estimated the proportions of distinct immune cell subpopulations in our RNA-seq data using CIBERSORT and the frequencies of different B-cell subsets (naïve and plasma cells) were found to be higher in normal tissue compared to tumor (Supplementary Fig. 1d). We also determined that unswitched memory B cells could be a predominant subset present within the population of proliferating tumor-infiltrating B cells (TIL-Bs; Supplementary Fig. 1e–f). In contrast with the previously described higher proliferating B-cell densities in peritumoral regions than in adjacent normal tissue, the total density of TIL-Bs and proliferating TIL-Bs in tissue mass was lower in tumor tissue than normal tissue (Supplementary Fig. 1g–h).

### Combined prognostic value of intratumoral HMGB1 expression and peritumoral proliferating B-cell counts for ESCC patients

**Fig. 2:**
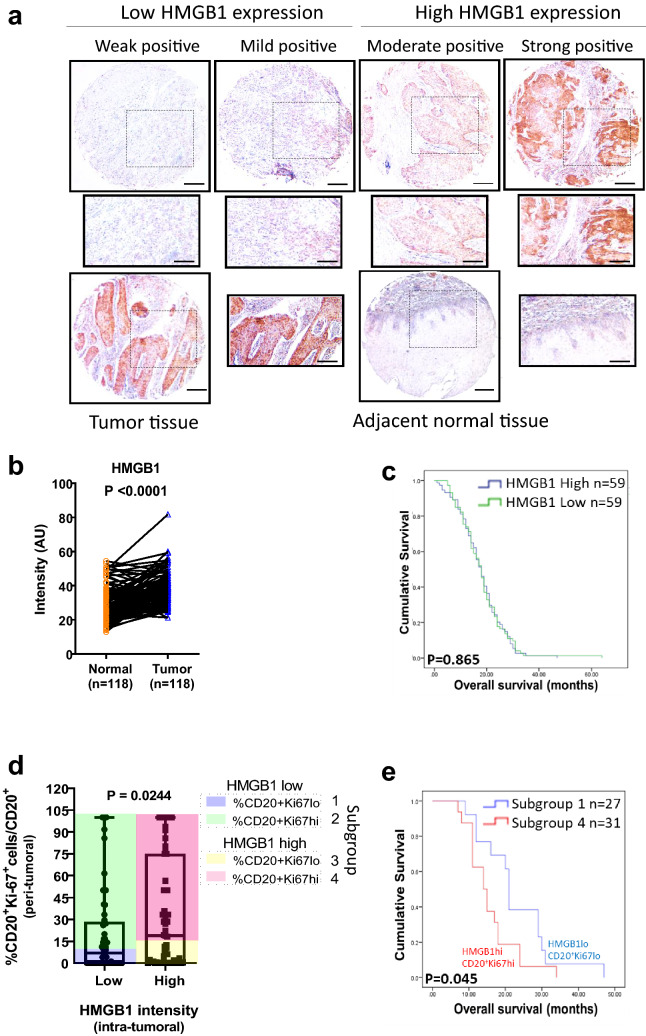
Combined evaluation of the intratumoral HMGB1 expression and peritumoral proliferating B cells can predict overall survival. **a** Immunohistochemical staining of HMGB1 in ESCC tumor and adjacent normal tissue. Scale bar: 200 μm (100 μm in the enlarged view). **b** Quantitation of the intensity (AU) data of HMGB1 expression by ImageJ. Specimens were categorized into four classes: weak, mild, moderate, and strong, according to staining intensity. **c** Kaplan–Meier curves with log-rank tests for overall survival (OS) according to HMGB1 expression (with the median fold change T/N as the cut-off). **d** Patients were divided into four subgroups (1, 2, 3, 4) among 74 cases showing peritumoral CD20^+^ cells. Subgroups were divided according to both intratumoral and peritumoral compartments: Subgroup 1: HMGB1 and CD20^+^Ki67^+^ counts were both low (blue). Subgroup 2: CD20^+^Ki67^+^ count was high (CD20^+^Ki67^+^hi) but low HMGB1 (green). Subgroup 3: CD20^+^Ki67^+^ count was low (CD20^+^Ki67^+^lo) but high HMGB1 (yellow). Subgroup 4: HMGB1 and CD20^+^Ki67^+^ counts were both low (red). **e** The OS of subgroup 4 (HMGB1hi CD20^+^Ki67^+^hi; red) was significantly shorter than subgroup 1 (HMGB1loCD20^+^Ki67^+^lo; blue)

Tumor-derived HMGB1 has been previously reported to resuscitate macrophage function in patients with ESCC [36]; we here aim to assess the clinical relevance of HMGB1 in B cells. We performed chromogenic staining of two TMAs incorporating total 125 cancer cases. Archival tissues from a total of 118 paired tumor/adjacent normal cases were selected to evaluate HMGB1 expression (Supplementary Fig. 2a). As expected, HMGB1 was detected in the intratumoral locations of tumor samples (Fig. 2a). We performed quantitative scoring based on HMGB1 staining intensity by Fuji software and found that HMGB1 expression was significantly higher (*p* < 0.001) in tumor relative to normal tissues, representing about 86% (102/118) of all ESCC-paired cases (Fig. 2b and Supplementary Fig. 2b). From patients with tumor RNA-seq data and an additional validation set of 38 unpaired ESCC samples, we further confirmed that HMGB1 mRNA expression was frequently upregulated in tumor tissues compared to the non-tumor counterpart (Supplementary Fig. 2c, d, respectively).

To investigate whether the presence of HMGB1 within the tumor nest could have an impact on predicting patients’ survival, we stratified patient samples into low or high variance groups. Using the median fold change in HMGB1 expression as the cut-off point (Tumor to Normal ratio, T/N = 1.374), patients were divided into a high HMGB1 expression group (T/N fold change > 1.374) and a low HMGB1 expression group (T/N fold change < 1.374). The Kaplan–Meier curves showed that HMGB1 expression was not an independent prognostic factor for overall survival (OS) in ESCC (*p* = 0.865, Fig. 2c). Also, age, sex, pathologic T and N stages, and tumor differentiation showed no significant correlation with HMGB1 expression (Supplementary Table 1).

We next evaluated the relationship between peritumoral proliferating B cells and intratumoral HMGB1 expression and their impact on patients’ survival. We divided the density of peritumoral proliferating B cells and intratumoral HMGB1 expression into groups of high/low based on the medians as cut-offs and observed a significant correlation (*p* = 0.0244) between these two variables (Fig. 2d). As shown in Fig. 2d, a high proportion of proliferating B cells and high expression of HMGB1 were found in 49% (62/125) and 53.6% (67/125) of cases, respectively. Of the cases that were highly positive for proliferating B cells, 24.8% (31/125) were also highly positive for HMGB1. A positive association was observed between intratumoral HMGB1 and peritumoral proliferating B cells, but not with total B cells density (Supplementary Fig. 2e). Using these two markers (HMGB1 high/low and proliferating B cells high/low) in combination, patients were divided into four groups (Supplementary Table 2). There was no significant difference between the groups with respect to their clinicopathologic characteristics. Strikingly, by Kaplan–Meier analysis, the OS of cases that were highly positive for both proliferating B cells and HMGB1-expressing tumor cells was shorter than for those with low expression of both factors (*p* = 0.045; Fig. 2e). Overall, the findings suggest that intratumoral HMGB1 may interact with peritumoral proliferating B cells to modulate ESCC biology.

### Cancer-derived HMGB1 promotes B-cell proliferation and migration

**Fig. 3:**
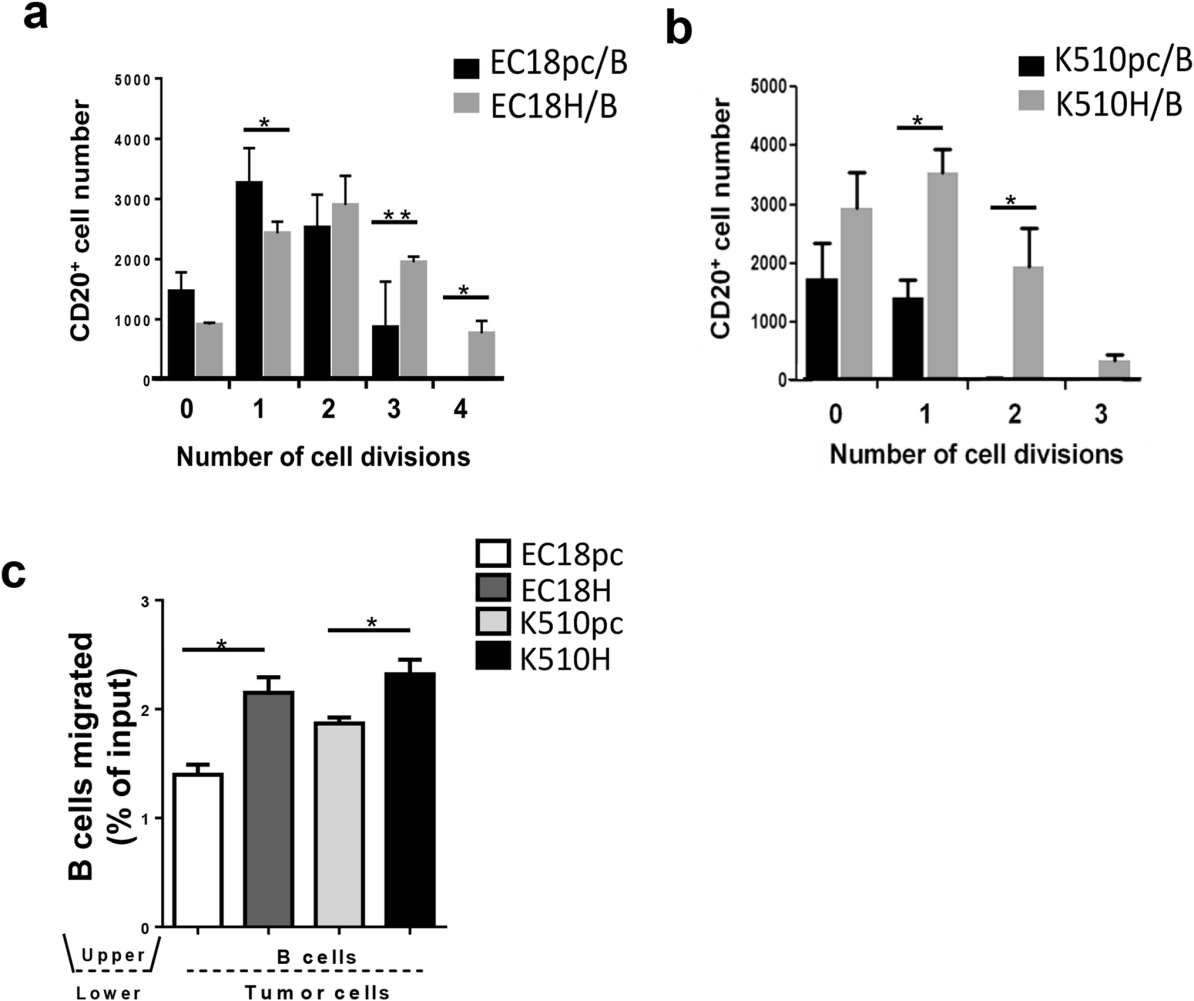
ESCC-derived HMGB1 promotes B-cell proliferation and migration. **a, b** CFSE-labeled B cells were pre-stimulated with IL-4 and IgM, washed, and co-cultured with HMGB1-overexpressing tumor cells (EC18H, K510H)/empty vector (EC18pc, K510pc) tumor cells. After 6 days, dye dilution profiles were obtained. **c** In vitro migration assays were performed using transwell chambers containing HMGB1-overexpressing tumor cells (EC18H, K510H)/empty vector (EC18pc, K510pc)-transfected tumor cells at the bottom and isolated B cells in the upper chamber and subjected to a 3-h migration period. Results are expressed as mean SEM of three independent experiments (*p < 0.05; ** p < 0.01)

To understand how B cells are regulated within the HMGB1-enriched tumor nest, we initially investigated if HMGB1 binds to the surface of B cells. We found that HMGB1 is bound to the surface of B cells, with an average Mander’s overlap coefficient of 0.914 and Pearson correlation coefficient of 0.79 (Supplementary Fig. 3a). We next provided evidence that HMGB1 acts directly as a proliferative signal for B cells (Supplementary Fig. 3b). These findings prompted us to examine the functions of cancer-derived HMGB1 on B cells. Stable cell lines overexpressing HMGB1 were established using the EC18 and K510 cell lines (thereafter referred as EC18H and K510H; Supplementary Fig. 3c–e) and co-cultured with peripheral blood mononuclear cells (PBMC) or freshly isolated CD20^+^ B cells (Supplementary Fig. 3f). The carboxyfluorescein succinimidyl ester (CFSE)-labeled PBMC (Supplementary Fig. 4a) and B cells (Fig. 3a–b and Supplementary Fig. 4b) were co-cultured with HMGB1-overexpressing (EC18H and K510H) or vector control (EC18pc and K510pc) cells for 6 days. We observed that the proliferation rate of B cells co-cultured with HMGB1-overexpressing tumor cells was higher than that of their vector control cells. Similar findings, although to a lesser extent, were observed on B cells in co-culture with tumor cells for 72 h, as revealed by Ki67 expression (Supplementary Fig. 4c). Our subsequent in vitro analyses therefore utilized 6 days co-culture systems to further characterize the mechanistic link between B cells and cancer-derived HMGB1, as well as their potential roles in orchestrating tumorigenesis and progression in ESCC.

In the majority of clinical samples, B-cell aggregates were enriched peritumorally (i.e., in the stroma at the invasive border of tumor nests; Supplementary Fig. 4d). This may suggest that B cells are attracted toward HMGB1-expressing tumor front and we consequently focused on the chemotactic capacity of the B cells in response to HMGB1. Transwell experiments were used to study migration of B cells in response to recombinant HMGB1 (rHMGB1; Supplementary Fig. 4e–f) or in the presence of HMGB1-overexpressing tumor cells (Fig. 3c). Increased chemotactic response toward the presence of HMGB1 was observed in B cells, suggesting that cancer-derived HMGB1 could be an important factor favoring B-cell retention within the TME.

### HMGB1-chemoattracted B cells promote HUVEC angiogenesis in vitro

**Fig. 4:**
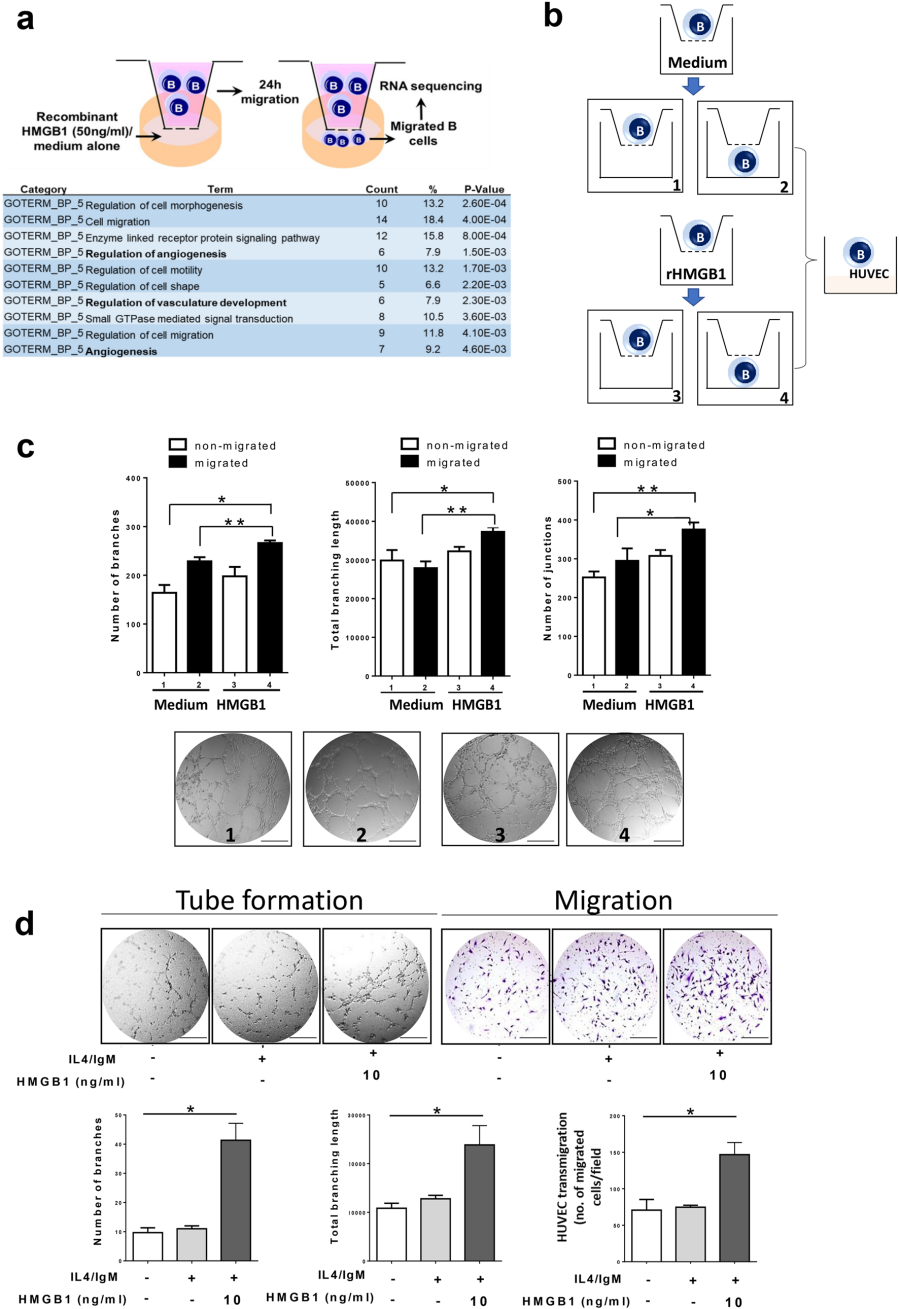
Transmigrated B cells in response to HMGB1 promote HUVEC angiogenesis in vitro. **a** Angiogenesis-related genes (bold) were found within the top 10 enriched biological processes among differentially expressed genes in B cells with enhanced migration upon recombinant HMGB1 treatment. **b** Schematic representation of the experimental design. Chemotaxis study on B cells using a transwell assay in the presence of recombinant HMGB1 (rHMGB1, 10 ng/mL) or medium alone (control). Subsequent analysis was performed on four subgroups. Subgroup 1: non-migrated B cells in transwell assay with medium. Subgroup 2: migrated B cells in transwell assay with medium. Subgroup 3: non-migrated B cells in transwell assay with rHMGB1. Subgroup 4: migrated B cells in transwell assay with rHMGB1. **c** Matrigel tube formation assay was performed to evaluate the angiogenic ability of HUVECs by overlaying with the migrated or non-migrated B cells. Representative micrographs images are shown at 200 × magnification. The number of branches/junctions/total branching length per high-power field was analyzed, and the data represent the means ± SEM of triplicates (*p < 0.05; **p < 0.001). **d** The conditioned medium (CM) was collected from B cells after exposure to rHMGB1 (10 ng/mL) for 24 h. HUVECs were incubated with CM for 6–8 h. Representative micrographs of the transwell and Matrigel tube formation assay (magnification 200 ×). The number of junctions/total branching length and transmigrated HUVECs per high-power field was analyzed, and the data represent the means ± SEM of triplicates (*p < 0.05; **p < 0.001). Scale bar: 200 µm

To analyze the impact of HMGB1 on migratory B cells, RNA sequencing on B cells migrated toward rHMGB1 or a medium control revealed that 76 genes were differentially expressed, of which 66 genes were upregulated. Among those 66 upregulated genes, the gene ontology analysis outcomes confirmed cell migration as one of the central processes for the HMGB1-educated migratory B cells. Most notably, angiogenesis was an unexpected category from the top ten enriched biological processes identified in enhanced migrated B cells, based on biological processes level 5 (Fig. 4a).

Next, we investigated the functional roles of HMGB1-transmigrated B cells on endothelial cells (ECs). Human umbilical vein endothelial cells (HUVECs) were overlaid on Matrigel and cultured with migrated or non-migrated B cells toward rHMGB1/medium alone, and the angiogenic ability of HUVECs was examined by tube formation and migration assays (Fig. 4b). As shown in Fig. 4c, enhanced EC capillary tube and network formation were observed on the Matrigel containing migrated B cells toward medium containing rHMGB1 (subgroup 4), as compared to medium alone (subgroup 2; increased by 1.62-, 1.33-, and 1.49-fold, with respect to number of branches, junctions, and total length of segments). To exclude the effect of direct contact between HUVECs and B cells on angiogenesis, HUVECs were incubated with conditioned medium (CM) from B cells pre-treated with/without rHMGB1. Figure 4d reveals that CM from rHMGB1-pre-treated B cells enhanced HUVEC angiogenesis in vitro. Also, no significant difference was observed in activated B cells without the stimuli of HMGB1, suggesting that only activated B cells in response to HMGB1 provoke tumor angiogenesis.

### Cancer-derived HMGB1 polarizes B cells to a proangiogenic phenotype

**Fig. 5:**
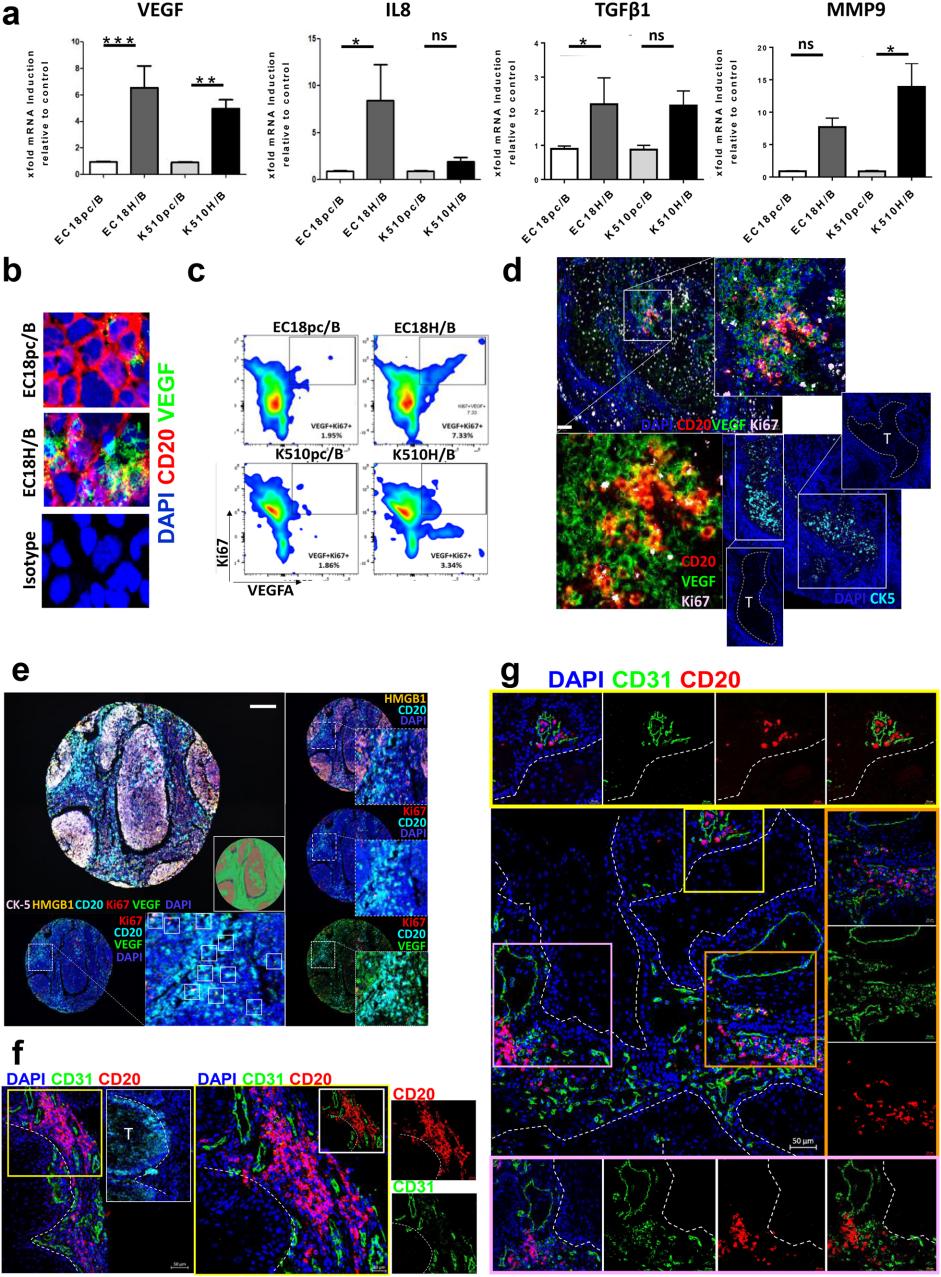
Tumor-derived HMGB1 drives B cells toward a proangiogenic profile **a** qRT-PCR analysis of the relative expression of angiogenic gene expression by B cells upon co-culture with HMGB1-overexpressing tumor cells (EC18H/K510H) or vector control (EC18pc/K510pc). Results are expressed as mean ± SEM of three independent experiments using each cell line. The fold difference relative to baseline expression was calculated by setting the gene expression in vector cell line as 1. Data were normalized using mammalian β-actin as an endogenous control gene. Results are expressed as the difference in threshold cycle (ΔCt), normalized to β-actin. *P ≤ 0.05, *** p < 0.001 relative to control. **b** B cells co-cultured with HMGB1-overexpressing or control tumor cells were isolated, cytospin followed by staining with antibodies against CD20 (1:50; red) and VEGF (1:80; green). Cell nuclei were counterstained with DAPI (blue) (magnification 400 ×). **c** Flow cytometric analysis of Ki67 and VEGF expression in B cells upon co-culture. **d** A representative example of ESCC tumor tissue demonstrating B cells (CD20, red) positive for VEGF (green) and Ki67 (pink). Scale bar: 100 µm. Higher magnification of the three-color overlay with (upper right) and without (bottom left) nuclei stain (DAPI, blue). Staining of cytokeratin-5 (CK-5; turquoise; bottom right) identified tumor region (labeled as “T”). **e** Representative TMA core with five markers (CK-5, HMGB1, Ki67, VEGF, and CD20). The image acquisition of all markers occurs simultaneously. Select combinations of markers are displayed. White inset indicative of Ki67^+^VEGF^+^-producing B cells. Scale bar: 100 µm. **f, g** B cells (CD20, red) accumulate around microvessels (CD31, green) in human ESCC tumors. Magnified insets with representative region of individual markers (or select combinations of markers) are shown on the side. The white broken line shows the area of tumor regions or labeled as “T.” Scale bar: 50 µm

We demonstrate here that HMGB1-conditioned B cells promote angiogenesis in HUVECs in vitro. However, the TME is complicated. We therefore further investigated whether ESCC-derived HMGB1 could drive B cells into a proangiogenic state. To exclude the effect of cell contact, B cells were co-cultivated with tumor cells via transwell assay. qRT-PCR analysis of genes implicated in angiogenesis process demonstrated that angiogenic factors, VEGF, IL8, TGFβ, and MMP9 were increased in the B cells co-cultured with HMGB1-overexpressing tumor cells as compared to control cells (Fig. 5a). VEGF is an important angiogenic factor and B cells are the possible source of this growth factor. In fact, immunocytochemical analysis (Fig. 5b) revealed upregulated VEGF production in B cells when co-cultured with HMGB1-overexpressing tumor cells.

Next, we performed flow sorting according to CFSE signals and confirmed that low CFSE signal (high proliferative signal) attributed to increased VEGF expression in B cells (Supplementary Fig. 5a). Quantification of VEGF and Ki67 expression by FACS in B cells confirmed the increased frequencies of proliferating VEGF^+^ B cells upon co-culture with HMGB1-overexpressing tumor cells (Fig. 5c and Supplementary 5b). Accumulation of Ki67^+^VEGF^+^ B cells was observed in the peritumoral area of ESCC tissue (Fig. 5d). Further analysis revealed that 51.2% (22/43) of the cases showed peritumoral proliferating VEGF^+^ B cells (Fig. 5e and Supplementary 5c), where the proportion of VEGF^+^ B cells subsets in total B cell counts was 56.3%, in which 14.3% was proliferative cells. Importantly, when CD31^+^ blood vessels are present, CD20^+^ B cells tended to accumulate around blood vessels rather than distributed evenly throughout tumor tissues, with the majority of B cells being < 10 µm from a blood vessel (Fig. 5e–f and Supplementary 5e). We considered two possible explanations for the close proximity of B cells to vasculature: 1) blood vessels are an important route for B cells trafficking to tumor sites or 2) B cells within the TME are critical for, or associated with, angiogenesis.

### HMGB1 secreted by tumor is required for VEGF-mediated proangiogenic effects in B cells

**Fig. 6:**
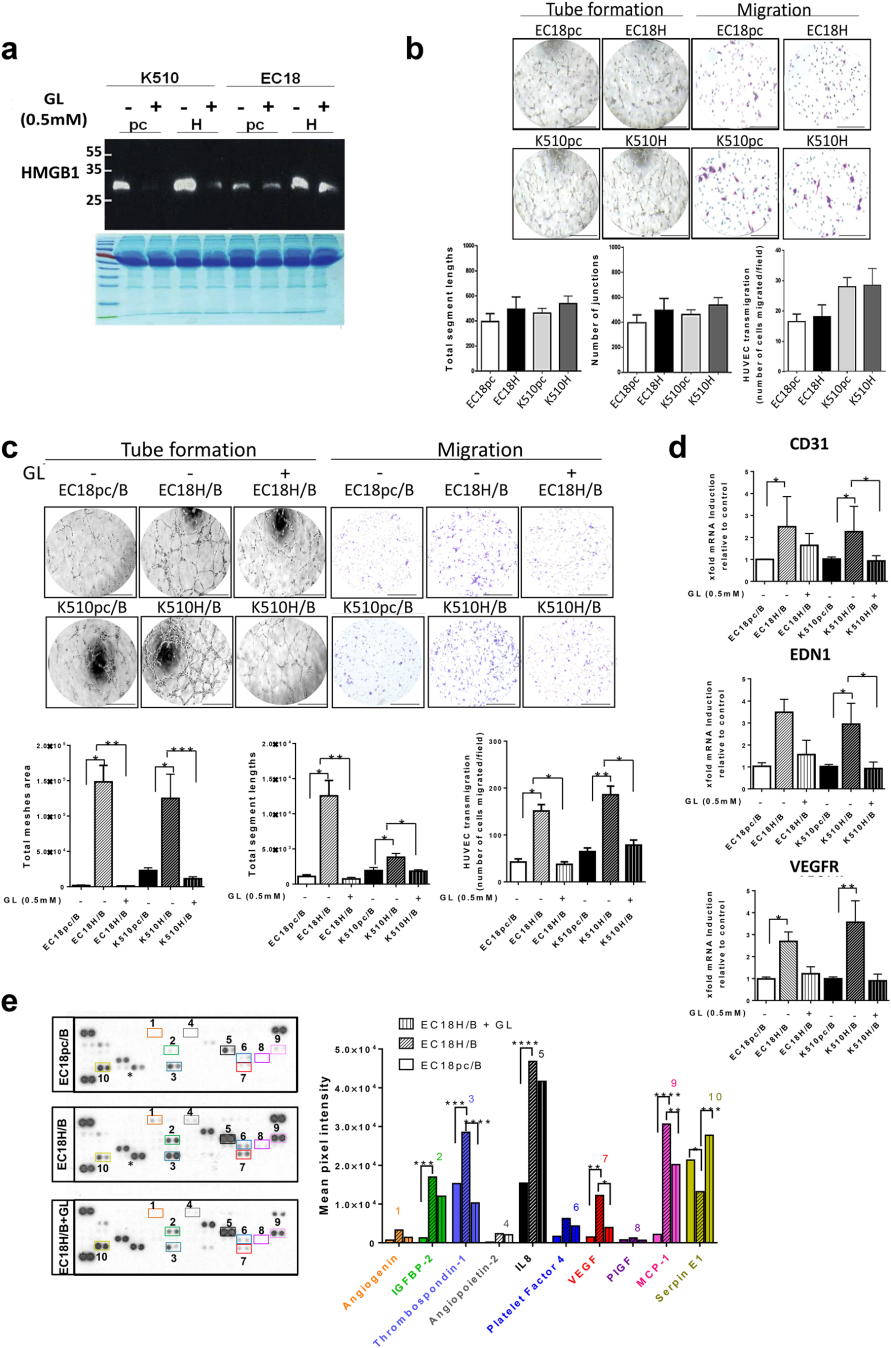
Glycyrrhizin suppresses tumor angiogenesis in HMGB1-overexpressing tumor/B-cell co-culture. **a** Cell supernatants collected from tumor cells treated in the presence ( +) or absence (-) of glycyrrhizin (GL, 0.5 mM) were subjected to western blotting for detecting HMGB1 soluble protein. Coomassie brilliant blue-stained gels were used to confirm equal loading. **b** Analysis of endothelial HUVECs cells migration and tube formation from conditioned medium (CM) from tumor cells overexpressing HMGB1 (EC18H, K510H) or vectors control (EC18pc and K510pc). HMGB1-expressing cells do not show any potentiate effect on HUVEC cell migration and differentiation (tube formation) when compared to vector cells (control). **c** Incubation of HUVECs with CM collected from co-cultures (EC18pc/B and K510pc/B vs. EC18H/B and K510H/B) in the presence ( +) or absence ( −) of GL (0.5 mM). HUVEC migration and tube formation were counted by ImageJ. Scale bar: 200 µm. **d** qRT-PCR analysis of the relative expression of VEGFR, CD31, and EDN1 in HUVECs after 24-h incubation with CM from the co-culture with ( +) or without ( −) GL. **e** The representative images of cytokine antibody array (left) and comparison of mean pixel density measurement among cytokines in collected CM of tumor/B-cell co-cultures in the presence or absence of GL (right). The arrays were quantified using ImageJ. Values are normalized to reference spots on the membranes (top left, top right, and bottom left). **d** Results are representative of three different experiments. *p < 0.05, **p < 0.01, ***p < 0.001 relative to control

The striking observation that soluble HMGB1 derived from tumor cells mediates the production of proliferating VEGF^+^ B cells, prompted us to ask if suppressing HMGB1 could interfere with this proangiogenic phenotype. We challenged this hypothesis by studying the crosstalk among tumor, B cells, and HUVECs through the use of CM. In brief, we first neutralized the secretion of HMGB1 from tumor cells by a direct HMGB1 antagonist, glycyrrhizin (GL; Fig. 6a). We found no angiogenic ability of HUVECs in the absence of B cells (Fig. 6b), suggesting that HMGB1 derived from ESCC cells alone was not sufficient to elicit tumor angiogenesis in vitro. We next analyzed the effect after co-culture of tumor with B cells in the presence or absence of GL. The administration of GL significantly inhibited capillary-like tube formation, including total mesh area (99.26% and 90.90% reduction in EC18H/B and K510H/B, respectively) and total length of segments (94.14% and 52.36% reduction in EC18H/B and K510H/B, respectively) (Fig. 6c). To better mimic in vivo blood vessel formation, we conducted HUVEC spheroid sprouting assays in 3D culture (Supplementary Fig. 6a). The results showed that the average sprout length in HUVECs treated with CM from co-culturing B cells with HMGB1-overexpressing tumor cells at 72 h was significantly longer (3.716 ± 5.062 μm/spheroid) than in those treated with control medium (1.692 ± 2.535 μm/spheroid). The spheroid sprouting abilities of HUVECs were suppressed by the presence of GL (Supplementary Fig. 6b,c). Likewise, CM from co-culturing B cells with GL-treated HMGB1-overexpressing tumor cells significantly reduced HUVEC transmigration via transwell assay (77.42% and 52.03% reduction in EC18H/B and K510H/B, respectively; Fig. 6c) and live-cell imaging (Supplementary Fig. 6d). In addition, qRT-PCR analysis revealed that the expression of genes implicated in vascular growth (*CD31*, *VEGFR*, and *ENDOTHELIN-1*) was induced in HUVECs after incubation with CM from EC18H/B and K510H/B co-cultures. This effect was dramatically reversed by the addition of GL (Fig. 6d). The observed angiogenic behavior was specific for HMGB1 since comparable in vitro neutralizing efficiency was obtained with the administration of an anti-HMGB1 monoclonal antibody (anti-HMGB1-mAb; Supplementary Fig. 7a,b). Together, our data highlight the relevance of tumor cell-derived HMGB1 in inducing proangiogenic B-cells activities.

Next, we demonstrated that ERK and p38 MAP kinase were activated (by phosphorylation) and involved in the promotion of VEGF expression in B cells via tumor-derived HMGB1 and the induction of VEGF was attenuated by the presence of GL (Supplementary Fig. 7c). To validate these results, we investigated the angiogenic components in the CM after co-culture with HMGB1-overexpressing tumor cells, using human angiogenesis arrays. As shown in Fig. 6e, several angiogenic factors were induced when B cells were co-cultured with tumor cells, and their levels were further upregulated in co-culture with HMGB1-overexpressing tumor cells. Treatment with GL significantly suppressed the secretion of several proangiogenic factors, such as VEGF, thrombospondin-1, IL-8, and monocyte chemoattractant protein-1. By contrast, the anti-angiogenic factor serpin E1 was restored by GL. Finally, we assessed whether VEGF was the main angiogenic factor produced by B cells upon HMGB1 stimulation. To this end, we investigated the chemotactic migration and tube formation in HUVECs via VEGF blockade (Supplementary Fig. 7d). A significant reduction of HUVEC functions was observed upon anti-VEGF antibody administration (Supplementary Fig. 7e). These data unequivocally demonstrate that cancer-derived HMGB1-induced angiogenesis in B cells is dependent on VEGF.

### HMGB1/B-cell interaction increases vascular density and tumor growth in esophageal cancer in vivo

**Fig. 7:**
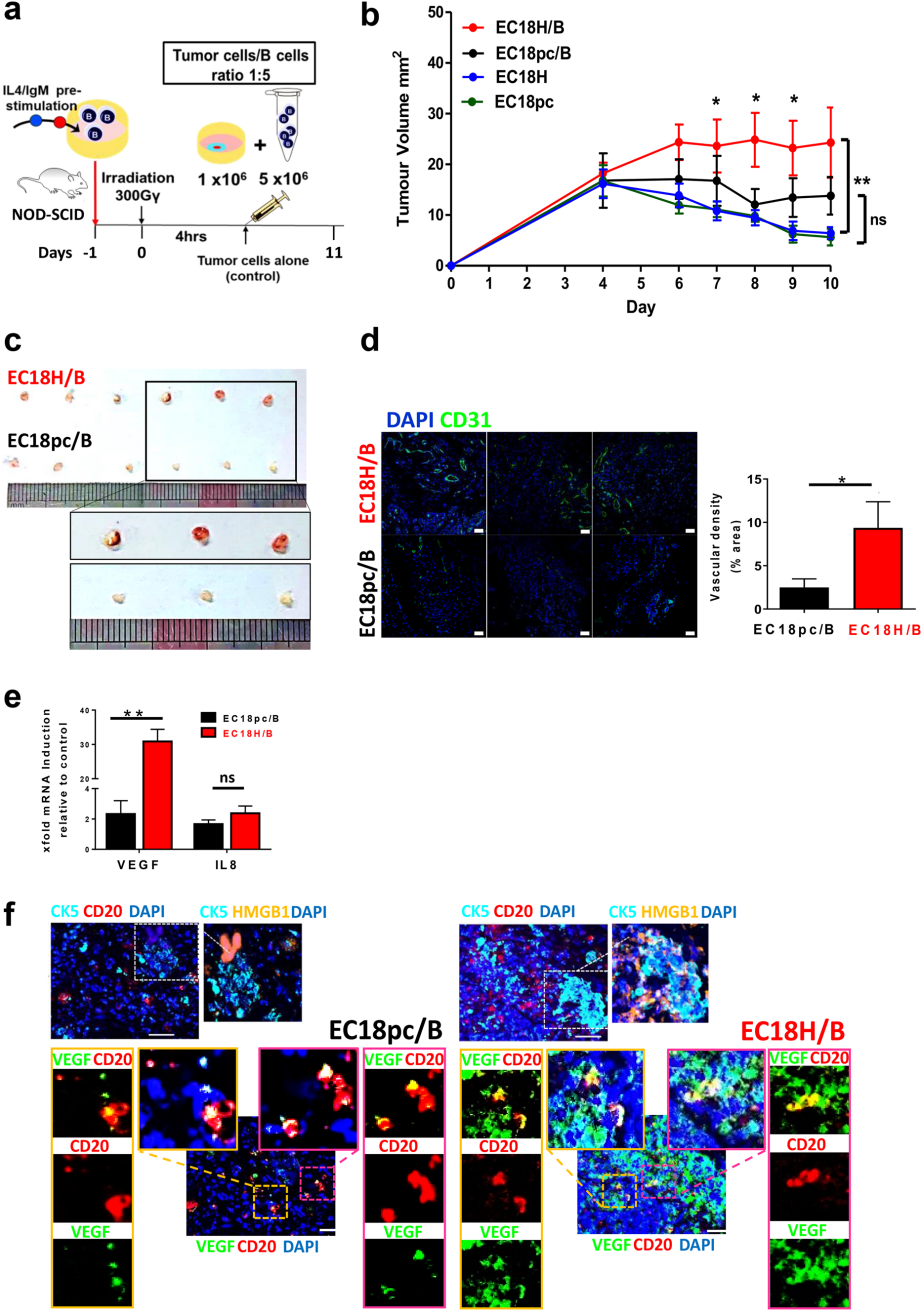
HMGB1/B-cell axis promotes tumor angiogenesis and growth. **a** Treatment scheme showing the timing of mice experiments (*n* = 6 per group). **b, c** NOD/SCID mice irradiated at 300 cGy and subcutaneously injected with tumor cells alone (EC18H vs. EC18pc; green vs. blue lines) or mixed with B cells (mixture ratio 1:5; EC18H/B vs. EC18pc/B; red vs. black lines). Tumor volumes of each group were compared (*p < 0.05; **p < 0.01). Tumors at the end point of measurement were isolated and photographed. **d** Tumor angiogenesis was assessed with immunohistochemical staining using an antibody against CD31 (green). Nuclei stained with DAPI (blue). Scale bar: 50 µm. The bar graph shows the quantification of vascular density. **e** qRT-PCR analysis of the relative expression of angiogenic gene expression (VEGF and IL8) in sorted B cells upon co-injection with HMGB1-overexpressing tumor cells (EC18H) or vector control (EC18pc) into NOD/SCID mice. Results are expressed as mean ± SEM of three tissues per group (sorted B cells from pooled tissues of two mice). The fold difference relative to baseline expression was calculated by setting gene expression in vector cell line as 1. **f** Three-color overlay of representative sampling of the simultaneously acquired markers (CK-5, HMGB1, Ki67, VEGF, and CD20) and enlarged insets of select combinations of markers are displayed (border). Image acquisition of select markers combination was shown. The high magnification shows more VEGF^+^ B cells (CD20 in red; VEGF in green, double-positive as yellow; bottom panel) in the peri-region of mice co-injected with EC18H/B cells mixture (tumor marker: cytokeratin-5 [CK5] in turquoise; HMGB1 in orange; top panel)

To better mimic the in vivo situation of microenvironmental interplay between HMGB1-expressing tumor cells and B cells, a mixture of the indicated cells was subcutaneously injected into NOD/SCID mice (Fig. 7a). As shown in Fig. 7b, tumor growth in HMGB1-overexpressing cells was similar to the vector control group (labeled as EC18H in blue and EC18pc in green, respectively). Compared with tumors in mice injected with EC18H tumor cells alone, higher tumor growth rate was observed in mice injected with B cells mixed with HMGB1-overexpressing tumor cells (labeled as EC18H/B in red). Moreover, the tumors of EC18H/B mice had more blood vessels, were larger in size (an average of 212% larger; Fig. 7c), and showed increased CD31 staining in tumor tissue compared with mice injected with vector cells mixed with B cells (labeled as EC18pc/B in black; Fig. 7d). To determine whether the different number of blood vessels attributed to tumor angiogenesis was due to the presence of proangiogenic B cells, the B cells from tumors were FACS sorted based on Epcam (epithelial cell marker), CD45 (immune cell marker), and CD20 (B-cell marker) (Supplementary 7f). We observed higher mRNA expression of VEGF and IL-8 in the B cells isolated from the EC18H/B group than the control vector EC18pc/B group (Fig. 7e). An enriched VEGF^+^ B-cell phenotype was further demonstrated by immunofluorescence (Fig. 7f).

**Fig. 8:**
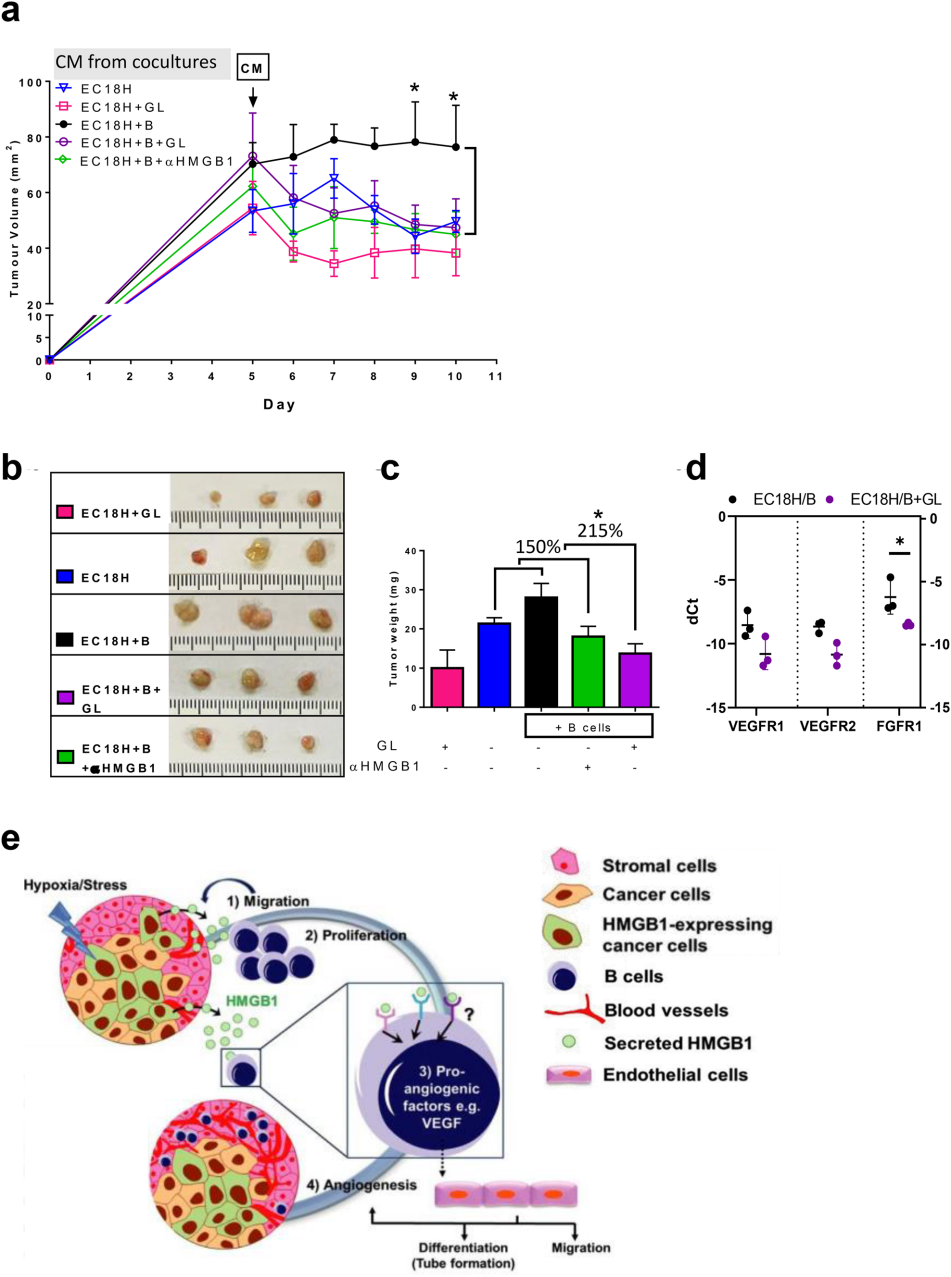
Tumor/B-cell secretome promotes angiogenesis in a HMGB1-dependent manner to fuel esophageal tumor growth in vivo. **a** NOD/SCID mice administered ESCC tumor by subcutaneous injection (*n* = 3 per group). Mice were either treated with concentrated CM collected from co-culture (black) in the presence of GL (purple) or αHMGB1 mAb (green) or concentrated CM from HMGB1-expressing cells alone in the presence (pink) or absence (blue) of GL for 5 days consecutively. Tumor volume change is shown. **b** Excised tumor and **c** tumor weight on day 7 after treatment are shown. **d** Relative quantification qRT-PCR to evaluate genes associated in angiogenesis (VEGFR1, VEGFR2, and FGFR1) in tumor. Results are expressed as the difference in threshold cycle (dCt). **e** A cartoon recapitulating the contribution of the endogenous balance between cancer-derived HMGB1 and its target B-cell activation is a modulator in regulating tumor angiogenesis and growth

To ascertain whether soluble factors, rather than direct cell–cell contact, contribute to angiogenesis, we focused on the CM collected from co-culture of EC18H/B in the absence or presence of GL/anti-HMGB1. As shown in Fig. 8a and b, mice in the anti-HMGB1 mAb group (labeled EC18H + B + αHMGB1 in green) and GL group (EC18H + B + GL in purple) had smaller tumor volumes and weights in comparison to their control tumors (labeled EC18H + B in black). Although treatment with GL alone (labeled EC18H + GL in pink) reduced basal tumor growth (compared with EC18H in blue), we showed that the addition of anti-HMGB1-mAb, and to a greater extent with GL, reversed the HMGB1-induced B-cell activation in tumor growth to a basal level (150% and 215% reduction in the effect of B cells mixed with EC18H, Fig. 8c). Consistently, loss of HMGB1 function in the GL-treatment group reduced the expression of genes associated with angiogenesis (Fig. 8d).

Collectively, our results suggest that B cells within the TME, being primed by HMGB1 released from cancer cells, migrate to the tumor. Here, they proliferate and activate a proangiogenic phenotype, leading to the activation of endothelial cells and ultimately tumor progression (our tentative synthesis is depicted in Fig. 8e).

## Discussion

An increasing number of studies demonstrates that the propensity for tumor progression is integrally associated with lymphoid cells within the TME and that the biological function between intratumoral and peritumoral immune cells may be different. Accordingly, peritumoral B cells have been reported as a positive prognosticator in NSCLC [42], whereas Nakamura et al. [43] observed no prognostic impact in the total number of intratumoral T cells. In contrast to T cells, considerably less is known about tumor-associated B cells. Both pro- and antitumorigenic activities have been ascribed to B cells and their roles in ESCC have not been satisfactorily evaluated so far. Based on the mIHC, FACS, and RNA-seq data generated in this study, we propose that local clustering of TIL-Bs may be a more distinctive feature than B-cell infiltrates in adjacent normal tissue. Moreover, the importance of spatial context and the nature of cellular heterogeneity of the TME, in terms of B-cell infiltrates in the intratumoral and/or peritumoral region, beyond overall densities, may provide additional contextual information relevant to the TME of ESCC.

In this timely context of clinical and translational research, the current study is the first in ESCC that has used precise quantitation of tumor cell–B-cell spatial relationships to predict OS. The three most striking findings of this study are: (1) ESCC is infiltrated with a significant amount of proliferating B cells at the peritumoral, but not intratumoral location. (2) The combination of overexpression of HMGB1 (intratumorally), together with high densities of proliferating B cells (peritumorally), was identified as an independent unfavorable prognostic factor. (3) Cancer-derived HMGB1 promoted the proliferation, migration, and proangiogenic characteristics in B cells. Subsequently, rich vascular secretome from the co-culture directly affected vessel ECs to foster ESCC tumorigenesis by promoting angiogenesis.

Our current focus on the spatial organization within the tumor architecture, which provides more precise biological information compared to total cell densities alone, may augment the prognostic value of immune infiltrates in human ESCC. A similar finding was recently reported in colorectal cancer, in which the density of CD8^+^ T cells at the tumor invasive margin was found to be more predictive of outcomes compared to the traditional Tumor–Node–Metastasis (TNM) scoring system [44] or microsatellite instability scores [45, 46]. It is of interest to note that despite we observed decreased total TIL-B ratios in ESCC tumor compared to their adjacent tissues, which is also described in a previous report [47], our data expand the observations by pinpointing the context-specific B-cell functions in ESCC. Moreover, our observation that more proliferating B cells were primarily located at the peritumoral region of ESCC tissues, suggesting a preferentially contact-independent communication between ESCC tumor cells and B cells. These data also imply that peritumoral proliferating B cells, albeit in low frequency, are sufficient to increase extrinsic factors in modulating tumor inflammation and unbalancing immune surveillance. Moreover, we observed slight discordance between mIHC as compared to flow cytometric analysis in proliferating B-cell numbers and percentages. One possible explanation is that the mIHC analysis selectively included the stromal area, enriched in B cells, whereas the flow cytometric analyses were performed on total tissue.

Aberrant HMGB1 signaling is associated with various human carcinomas. The upregulation of HMGB1 expression in tumor tissues was also observed in our ESCC cohort; however, our data showed that HMGB1 was not an independent prognostic factor for ESCC. This discrepancy could be due to the difference in assessment methods: other studies assessed the staining semiquantitatively by visual scoring by pathologist, whereas we utilized a digital image analysis method for more accurate quantification of HMGB1 staining. One critical question was then raised: What are the functions of these high levels of HMGB1 in tumor nest?

Since the immune-regulatory functions of soluble factors are increasingly recognized in cancers, we hypothesized that tumor-derived HMGB1 can be pivotal for influencing B-cell polarization during development of ESCC. An observation in OPSCC demonstrated that intra-epithelial B cells interacted with T cells via CXCL9 in the TME [10], while tumor killing capability of B cells was induced by IL-17A in ESCC [9]. Regarding HMGB1 and B-cell interactions, a recent study indicated that tumor-derived HMGB1 induced the expression of TIM1^+^ regulatory B cells in hepatocellular carcinoma [41]. Based on our work, ESCC tumor-derived HMGB1 assumes a strong influence in B cells. As HMGB1 can be actively secreted as well as passively released to emit a general alarm signal, co-culturing of B cells and ESCC cells in this study via transwell assays was used to mimic their microenvironmental interplay by assuring proper induction of function through soluble factors rather than direct cell–cell contact. We consistently observed increased levels of angiogenesis-related cytokines in the CM of these co-cultures, in particular upon co-culture with HMGB1-expressing tumor cells. We unveil strong evidence that soluble HMGB1 released from cancer cells can drive the proliferating B cells into a proangiogenic state for mediating VEGF-dependent angiogenesis. We also found that cancer-derived HMGB1 alone has no impact on tube formation or migration, further emphasizing the relevance of B cells in the control of tumor angiogenesis.

Notably, our in vivo studies with B cells co-injection with tumor cells in ESCC mice models fully corroborated the in vitro findings with respect to tumor growth and angiogenesis. In line with the regulation of angiogenic markers observed in B cells in vitro upon co-culturing, the animal experiments confirmed the modulation of several angiogenic markers, including VEGF and IL8, upon co-injection with HMGB1-expressing tumor cells to enhance their proangiogenic profiles in the TME. Furthermore, the discrete effect observed with respect to increased tumor growth and weight in mice injected with CM from co-culture with HMGB1-expressing tumor cells, highlighting that secretion of mediators between B cells and tumor cells could promote an overall effect on tumor progression. In the context of HMGB1 blockades, besides the HMGB1 antagonist GL, an anti-HMGB1 mAb neutralizing antibody was also tested. Despite GL’s anti-viral and anti-inflammatory effects, these two inhibitors displayed comparable in vitro and in vivo suppressive efficiencies in angiogenic activities. It was once again demonstrated that GL acts as a direct inhibitor of HMGB1 [48] and, consistent with a previous report [49], GL exerts similar effectiveness as anti-HMGB1 mAb. To the best of our knowledge, this report provides the first evidence that HMGB1 encountered within the TME closely interacts with B cells for the secretion of proangiogenic factors that promote angiogenesis. We are aware of the study limitation that the expression of human CD20^+^ B cells in the resected tumors could not be largely procured at the end point of measurement, possibly due to lack of T cells to support retained levels of injected human B cell. Nevertheless, the photographic images of resected tumor and immunohistochemical staining support the findings that the HMGB1/B-cell axis alters CD31 in established lesions. The in vivo results using CM from co-culture systems showed that the role in tumor growth suppression of GL/anti-HMGB1 mAb might be dictated by the presence of HMGB1, irrespective of B cells. However, both inhibitors could reverse the HMGB1-induced B-cell activation in tumor growth, indicating the importance of in situ ESCC-derived HMGB1–B-cell interactions in the protumor response.

Tumor-infiltrating B cells have been previously shown to produce antitumor antibodies/cytokines [11–13, 50]. Contrary to a report which demonstrated a proangiogenic phenotype in IgG4-switched memory B cells [16], we found that B-cell-specific gene expressions (i.e., *BACH2* and *PAX5*), rather than plasma cell (PC)-associated genes (i.e., *PRDM1*), were induced in B cells when co-cultured with HMGB1-overexpressing tumor cells (Supplementary Fig. 8a). The immunoglobulin ELISA, flow cytometric, and immunohistochemical analyses indicated that B cells co-cultivated with HMGB1-overexpressing tumor cells did not exhibit characteristics of class-switched and/or antibody-secreting phenotypes (Supplementary Fig. 8b–d). Supporting this, our RNA-seq data on three pairs of ESCC tissues showed that PC represented a rare population within the tumor tissues (Supplementary Fig. 1d). Thus, the enriched expression of HMGB1 may not participate in the commitment of B cells to the PC differentiation pathway in ESCC. The observed undefined proliferative B-cell subset documented with proangiogenic functions within the HMGB1-enriched TME of ESCC needs to be further examined. HMGB1 was originally described as a nuclear protein; extracellular HMGB1 is recognized as a prototypical DAMP, which promotes tumor progression [51]. One of the mechanisms for B-cell activation is binding to TLR-2, TLR-4, TLR-9, and RAGE, which mediate HMGB1-dependent activation. HMGB1 has been found to form an immune complex with DNA and activate B-cell responses via RAGE-dependent [52] or -independent interactions [53]. Moreover, HMGB1 can also interact with TLR-2 and CD36 for B-cell activation [54]. So far, there are 11 receptors reported for HMGB1 [55], the precise cell surface receptor(s) for HMGB1 interaction with B cells remains to be explored.

Collectively, this study provides an extensive analysis of B cells in the TME of ESCC, highlighting the prognostic significance of the pre-existing profile of HMGB1 and B-cell distribution inside and outside the tumor nest. Our results could have important implications for clinical therapeutic strategies. Since patients with high intratumoral HMGB1^+^ cells and high peritumoral proliferating B cells have a statistically significant shorter OS, this group may be considered for more intensive or novel treatment. Additionally, we showed that cancer-derived HMGB1 conditioned B cells could maintain a proangiogenic TME in ESCC. Our work explores the possible regulation of the HMGB1/B-cell axis in mediating ESCC progression. Future therapeutics may target pathological proliferating B cells as well as HMGB1 signals for anti-angiogenic in ESCC.

## Materials and methods

### Cell lines

ESCC cell lines KYSE140 (K140), K180, K410, K510, and K520 were obtained from DSMZ, the German Resource Center for Biological Material. ESCC cell lines EC18, HKESC1, and immortalized esophageal epithelial cell line NE1 were provided by Professors G. Srivastava and G.S. Tsao (The University of Hong Kong). HUVECs were provided by Dr Stephanie Ma (The University of Hong Kong). All experiments were done using endothelial cells between passages 3 and 8.

### Patients and samples

Four TMAs (two slides each for TMA-1 and TMA-2) composed of samples from a total of 125 ESCC patients from the surgical pathology archives of Linzhou Cancer Hospital (Henan, China) were used for histological staining. Of these, 89 paired ESCC and corresponding normal tissue samples from TMA-1 were used to evaluate B-cell spatial analysis by mIHC. One hundred and eighteen matched normal/tumor pairs from a consecutive slide of TMA-1 (*n* = 89) and TMA-2 (*n* = 29) were chosen to perform HMGB1 chromogenic immunohistochemistry. All tumor cases positive for B cells (*n* = 74 from TMA-1 and *n* = 51 from TMA-2) were chosen for cooperative biomarkers prognostic analysis. A summary of cases used for TMA is shown in Supplementary Fig. 2a.

Three paired ESCC tissues were used for RNA sequencing. An additional 38 paired ESCC tissues were used for qRT-PCR analysis. No patient in the study had received preoperative radiation or chemotherapy. Studies using human tissues were approved by the committees for ethical review of research involving human subjects of Zhengzhou University (Zhengzhou, China) and the Institutional Review Board of The University of Hong Kong/Hospital Authority Hong Kong West Cluster (HKU/HA HKW IRB).

Seven ESCC patients who had undergone upfront esophagectomy at the Department of Surgery, Queen Mary Hospital, Hong Kong were also recruited for tissue dissociation and subsequent flow cytometric analysis.

PBMC were isolated from healthy donors (buffy coats, Hong Kong Red Cross Blood Transfusion Service) for subsequent culture.

### Sample collection and processing

PBMC were isolated from healthy donors by Ficoll-Paque gradient (GE Healthcare) and centrifuged at 400×*g* for 25 min. The interface containing PBMC was carefully removed and cells were washed twice with PBS + 2 mM EDTA.

The tumor and normal tissue samples were processed into single-cell suspensions by mechanical disaggregation followed by enzymatic digestion using 1.5 μg/mL collagenase IV (Roche), 0.8 mg/mL dispase (Invitrogen), and 0.1 mg/mL DNase I (Sigma). Tissues were incubated in digestion medium at 37 °C for 30 min; released cells were collected and filtered, and the remaining tissue was further processed.

### Purification of B cells

PBMC were then re-suspended in chilled MACS buffer (PBS, 0.5% FCS and 2 mM EDTA), washed and incubated with CD20 microbeads (130-091-104; Miltenyi Biotec) for 15 min at 4 °C, and subsequently passed through magnetic separation columns (LS; Miltenyi Biotec). The bead-bound cells were collected as enriched, positively selected B cells. Purified CD20^+^ B cells were either re-suspended in FACS buffer for flow cytometry or in DMEM supplemented with 10% FCS, 50 IU/mL penicillin–streptomycin, and 10 mM HEPES buffer (Gibco/Invitrogen) for subsequent culture. Preparations were typically > 95% pure.

### B-cell chemotaxis

Directional migration of B cells was evaluated in Costar Transwell permeable polycarbonate supports (5 μm pores) in 24-well plates. HMGB1-overexpressing tumor cells/different concentration of recombinant HMGB1 (rHMGB1) were used to compare with parental cell lines/medium alone. B cells (0.25 × 10^6^ cells/ml) pre-treated with 100 ng/mL recombinant IL-4 and 5 µg/mL IgM for 24 h were washed, placed in the top chamber, and allowed to migrate for 4 h at 37 °C. After that, cells in the bottom chambers were collected, stained with FITC-labeled anti-CD20 antibody, and the number of CD20^+^ B cells was calculated by flow cytometry.

### ELISA

Supernatants from co-cultured cells were collected. Total IgG/M were detected using human IgG/IgM ELISA quantitation set (Bethyl Laboratories) according to the manufacturer’s instructions.

### B-cell proliferation

PBMC/purified B cells (1 × 10^6^/well) were labeled with 5 µM CFSE (Biolegend) in PBS/0.1% BSA for 8 min at 37 °C. Unbound dye was quenched by washing three times with ice-cold complete medium, followed by stimulation with 100 ng/mL recombinant IL-4 and 5 µg/mL IgM. 24 h later, pre-stimulated CFSE-labeled PBMC/B cells (1 × 10^4^) were washed and mixed with ESCC cell lines in 96-round bottom plate at effector-to-target ratios of 10:1. Proliferation of PBMC/B cells was monitored by CFSE partitioning 6 days post-co-culture. The total B-cell population from PBMC, stained with anti-CD20-APC antibodies, was analyzed with a FACSCanto II. In some experiments, pre-stimulated B cells were treated in the presence or absence of rHMGB1 (10 ng/mL) or anti-VEGF (10 µg/mL; Sino Biological), cells were collected for flow cytometric analysis, and conditioned medium (CM) was collected for subsequent functional assays.

### Collection of conditioned medium

CM was collected and centrifuged at 2000×*g* for 10 min. CM was either used undiluted for characterization by protein array or concentrated for an in vivo mouse model by centrifugation at 4000×*g* for 15 min at 13 °C, using ultrafiltration units (Amicon Ultra-PL 10, Millipore, Bedford, MA, USA). Filter units were used only once to avoid membrane saturation. Concentrated CM were then sterilized on 0.22 μm filters (Millipore), aliquoted, and stored at –80 °C until use.

### Matrigel tube formation assay

HUVECs were maintained in endothelial cell growth medium M200 (Invitrogen) supplemented with 2% FBS and endothelial cell growth supplements (LSGS Medium). 2 × 10^4^ cells were re-suspended in 100 µL co-culture CM (1:5; diluted with serum-free M200) and seeded in 96-well plates pre-coated with 50 µL of growth factor-reduced Matrigel (BD Bioscience). The cells were incubated at 37 °C for 6–8 h to allow for the formation of tube-like structures. Enclosed networks of tube structures from three fields were photographed randomly in each well and quantified using ImageJ analysis.

### Spheroid sprouting assay and imaging

Spheroids containing 6000 HUVECs were generated by incubating suspended cells in M200 in 96-well Corning® Spheroid Ultra-Low Attachment Microplates overnight, after which they were embedded into 3 mg/mL Matrigel for a fixed 3D cell culture. In brief, 70 µL hydrogel solution containing 3 mg/mL Matrigel and cells were dispensed into each well followed by 20 µL of CM. The plate was then warmed to 37 °C to induce gelation. Images of the spheroid within the polymerized gels were captured using InCell6500 (Perkin Elmer). Endothelial sprouts were characterized by measuring average branch lengths using Fiji distribution of ImageJ. Briefly, images were converted to 8-bit grayscale then converted to binary images with appropriate threshold values. Parameters for the binary mask, such as area and perimeter, were analyzed using the ‘Analyze Particles’ function. For end-point calculation, the areas covered by the spheroids were traced followed by ‘Skeletonize’ function of Fiji for analysis.

### Migration assay

Starved HUVECs (2 × 10^4^) were seeded in the top chambers of Transwell plates (8 µm pore size) in 400 µL of M200 medium without serum. The bottom chambers were filled with 400 µL co-culture CM (1:5 dilutions). After 24 h, cells were fixed, stained with 1% crystal violet, and counted. For gene expression assays, HUVECs were plated in 24-well plates (5 × 10^4^cell/well) incubated with CM (1:5 dilutions) for 8 h and 24 h. For the μ-slide chemotaxis assay (Ibidi), HUVECs were cultured on 2 µg/cm^2^ Matrigel-coated μ-slides and allowed to adhere for 3 h. One reservoir was filled with M200/2% FBS/LSGS and the second reservoir with the indicated 1:5 diluted CM collected from the co-cultures. Directional migration was assessed after 16 h and tracked with the aid of time-lapse microscopy.

### Angiogenesis antibody arrays

The relative levels of human angiogenesis-related proteins in CM were measured using a Human Angiogenesis Array Kit (R&D Systems Inc.). Aliquots of CM (500 µL) were added to the array and the results were analyzed with the ImageJ software.

### Immunohistochemistry (IHC) and immunocytochemistry (ICC)

#### Chromogenic IHC

Sections of 4 μm thickness were cut from FFPE tissue blocks. The slides were deparaffinized in xylene, rehydrated, and this was followed by an antigen retrieval step by heating at 95 °C for 45 min in citrate buffer (pH6). Endogenous peroxidase was blocked with peroxidase blocking reagent (Dako) followed by a non-specific binding protein block (Dako, X0909). For double staining, EnVision G|2 Doublestain System was used, according to the manufacturer’s instructions. Sections were then incubated with either mouse anti-human HMGB1 (1:400, ab18256, Abcam) and/or anti-human CD20 (1:50, Dako) and a species-matched isotype control overnight at 4 °C. Slides were then washed and secondary staining was performed with Dako REAL EnVision Detection System (K5007, Dako) and visualized with diaminobenzidine (DAB) according to the kit’s instructions.

#### Immunofluorescence IHC

After deparaffinization and blocking steps, sections were incubated with rabbit anti-human CD31 (1:100, ab28364, Abcam), mouse anti-human CD20 (1:50, Dako), rabbit anti-human CD20 (1:400 Thermo Fisher), mouse anti-human VEGF (1:80, MA-13182, Thermo Fisher), and a species-matched isotype control. Slides were then washed and secondary staining was performed with donkey anti-mouse-Alexa-555 or goat anti-rabbit-Alexa-488 in the case of the double stain for CD20/CD31 and EBI-3. For double staining of CD20/VEGF, goat anti-mouse-Alexa-555 or goat anti-rabbit-Alexa-488 was used. All slides were counterstained with DAPI and examined under Carl Zeiss LSM 700.

#### Immunofluorescence ICC

B cells were collected following recombinant HMGB1 incubation or co-culture with tumor cells and then washed twice with cold PBS cytospin. Cells were then fixed in methanol/acetone 1:1 followed by blocking and antibody incubation as described above.

#### Multiplexed IHC

For tyramide signal amplification (TSA) IHC staining, slides were first deparaffinized and rehydrated in serial passage through xylene and alcohol. Antigen retrieval was performed by microwaving the samples for 2 min 20 s with 100% power, followed by 20% power for 15 min and the slide was cooled for 20 min. Then, the sections were incubated with blocking solution, Biocare Medical Background Sniper supplemented with 2% BSA, for 15 min at room temperature. Slides were incubated with primary antibodies: Ki67 (1:50, Dako pH6), CD20 (1:10,000, EDTA pH9) and Cytokeratin-5 (1:10, Diva), VEGF (1:100, EDTA pH9) for 1 h at room temperature. Multiplexed TSA was visualized using performing a triplex (CD20 in Opal 650, CK-5 in Opal 570,Ki67 in Opal 690, and/or VEGF in Opal 570), followed by a 5-plex (addition of HMGB1 Opal 520). All multiplex TSA analyses were performed by repeating staining cycles in series, microwaving in between each cycle and at the end of the multiplex TSA. Slides were then counterstained with DAPI for 5 min and mounted with VECTASHIELD.

### Digital image acquisition and analysis

TMA sections were digitally scanned at an absolute magnification of × 20 using the Vectra 3.0 Vectra Polaris imaging system (Akoya Biosciences) and analyzed with inForm Tissue Finder software (Akoya Biosciences). Multispectral images were unmixed using the spectral libraries built from images of single-stained slides. Firstly, 15 TMA cores were selected to train machine learning algorithms for tissue segmentation, cell segmentation, and cell phenotyping, which were later applied on the whole TMA cohort. The software was first trained to segment tissue by manually to segment tumor tissues into carcinoma, the intra- (epithelial), and stromal, the peri-(non-epithelial), areas based on tumor marker, cytokeratin-5 (CK-5). To detect immune cells, an algorithm was designed based on pattern recognition that quantified CD20, VEGF, and Ki67 cells. After which the distribution of immune cells was analyzed and cell segmentation was based on the nuclear DAPI stain but assisted using membrane CD20 staining. Training sessions for tissue segmentation and phenotype recognition were carried out repeatedly until the algorithm reached the level of confidence recommended by the program supplier (at least 90% accuracy) before performing final evaluation. Each scanned image was examined by one observer under the supervision of an experienced pathologist. The area of each tissue category, carcinoma, and stroma was evaluated to assess the density of lymphocytes, represented by (number of lymphocytes)/(pixel area mm^2^) in each tumor cores. Spatial relationships between cellular phenotypes CD20^+^ and CD31^+^ cells in the peritumoral area were determined using the phenoptrReports package (Akoya Biosciences). Then, the distribution between CD20^+^ and CD31^+^ cells was identified in consecutive 10 µm steps (distance classes) within 200 µm.

HMGB1 immunohistochemistry was quantified in tissues using ImageJ (Ver. 1.52b). As tumorous samples consisted of mixed cell types including tumor cells and immune cells, image segmentation was first performed to isolate tumor and non-tumor regions. This allowed us to determine the regions and boundaries between the tumor cells and surrounding stroma. A supervised training of the Trainable Weka Segmentation (TWS) from Fiji (ver. 3.2.3) [56] was performed. A training set of five randomly selected 1604 × 1604 pixel images consisted of features from three classes: tumor, non-tumor, and background. The feature set used for training included a total of 80 attributes. A multithreaded implementation of random forest classifier with 200 trees and two features per node was used to build the model. The model was then applied to all images for classification. Representative results of segmentation are presented in Fig. 1A. Quantification of staining intensity of HMGB1 in tumorous or normal tissue were performed by first obtaining average pixel intensity of the identified tumor region in the tumorous samples or average intensity of the healthy tissue samples, respectively, followed by background subtraction.

### Establishment of stable HMGB1 overexpression cell lines

K510 and EC18 ESCC cell lines were maintained in RPMI and DMEM supplemented with 10% FBS and antibiotics, respectively. To generate stable HMGB1-overexpressing clones, cells were transfected by lipofectamine 2000 with pcDNA3.1 vector. Stable cell lines were selected by adding 500 µg/mL G418 for EC18 tumor cells and 100 µg/mL for K510 tumor cells.

### Co-culture of tumor and B cells

ESCC overexpressing the HMGB1 gene (K510H or EC18H) or vector (K510pc or EC18pc) were seeded into a 24-well plate (5 × 10^4^/mL/well) and used for co-culture experiments at 80% confluence. CD20^+^ B cells (1 × 10^6^ cells) were pre-treated with 5 µg/mL IgM and 100 ng/mL IL-4 for 24 h, washed, and co-cultured with the relevant ESCC cell line (5 × 10^4^ cells/well) through microporous cell inserts (1 µm pore size) in a 24-well plate for 6 days. Where indicated, the HMGB1 inhibitor GL (0.5 mM; Sigma), anti-HMGB1 monoclonal antibody (αHMGB1; 10 µg/mL; Arigo), or DMSO were added to ESCC cell lines before co-culture.

### Quantitative real-time PCR (qRT-PCR)

Total RNA was isolated using the RNeasy Mini kit (Qiagen). RNA was reversed transcribed (Takara) into cDNA, which served as a template for the amplification by qRT-PCR using the SYBR Green gene expression assay (Applied Biosystems 7900). Relative quantification was measured using the comparative Ct (threshold cycle) and the Ct values were normalized to β-actin or GAPDH, where appropriate. Primers used are shown in Supplementary Table 3.

### RNA-Seq and transcriptomic expression analysis

CD20^+^ B cells isolated from two healthy donor PBMC samples were subjected to a migration study for 24 h. B cells that migrated toward recombinant HMGB1 or medium only as a control were collected and RNA was isolated for sequencing using the SMART-Seq™ v4 Ultra™ Low Input RNA Kit. PCR products were amplified and sequenced on an Illumina HiSeq™ 2500 platform by Novogene (Beijing China). High-quality clean reads from all two samples were merged together and mapped to the reference sequence. To determine the biological significance of the differentially expressed genes, which were defined as genes with log_2_ expression fold change ≥ 0.5,or ≤ − 0.5, functional classification and gene enrichment analysis were performed using GO Term (Biological Process level 5) with DAVID Bioinformatics Resources. Top ten highly enriched functional categories were listed and arranged in descending order of p-value of enrichment.

### Flow cytometry

B cells collected from the transwell of co-cultured systems were washed and re-suspended in FACS buffer (PBS, 0.5% BSA, and 2 mM EDTA). Cells were stained with antibodies to the surface markers CD19, CD27, and CD38 (BD Biosciences) for 30 min on ice, followed by intracellular staining for Ki67 and VEGFA (BD Biosciences) using a BD Cytofix/Cytoperm kit. Antibodies were diluted FACS buffer for surface staining and in BD Perm/Wash buffer for intracellular staining. Cells were analyzed on a NovoCyte Quanteon and the data were analyzed using FlowJo Software.

### Western blot

Anti-phospho-ERK (1:500, sc-7383, Santa Cruz Biotechnology), anti-ERK (1:500, sc-94, Santa Cruz Biotechnology), anti-phospho-p38 (1:1000, 9211, Cell Signaling Technology), anti-p38 (1:1000, 9212, Cell Signaling Technology), anti-HMGB1 (1:1000, ab18256, Abcam), anti-beta-actin (1:5000, ab6276, Abcam), and anti-VEGF (1:1000, ab46154, Abcam) were used as primary antibodies. Cells were lysed in RIPA buffer containing protease and phosphatase inhibitor on ice for 45 min and collected by centrifugation at 16000×*g* for 15 min at 4 °C. Protein concentrations were measured by the bicinchoninic acid protein assay kit (Pierce). Cell lysates containing 30 µg of total protein were separated by 10% SDS-PAGE and subsequently transferred to nitrocellulose membranes. The membranes were subsequently probed with the indicated antibodies and proteins were detected using the ECL Plus Western Blotting Detection System (GE Healthcare).

### In vivo tumor experiments

For co-implanting tumor cells with B cells, 6-week-old NOD/SCID mice were irradiated at 300 cGy, then 4 h later, purified CD20^+^ B cells pre-treated with IgM and IL-4 were mixed with tumor cells (1 × 10^6^ HMGB1-overexpressing/empty vectors in 100 µl of PBS, *n* = 6 per group) in growth factor-reduced Matrigel (BD Biosciences) at a 5:1 ratio, and then implanted subcutaneously into mice under anesthesia. B cells or tumor cells alone served as controls. Tumor growth was monitored every 2 days for the indicated time. Excised tissues were divided into portions for mIHC and FACS. To increase the yield of B cells, two tissues from the same group were pooled together for enzymatic dissociation and B cells were sorted based on Epcam (epithelial cell marker), CD45 (immune cell marker), and CD20 (B-cell marker), followed by qRT-PCR analysis. A loss-of-function analysis was performed by subcutaneous injection with 5 × 10^6^ tumor cells. Five days later, mice were treated with 50 μL CM collected from co-culture by intratumoral injection for consecutive 5 days. The control group received CM from co-culture in the absence of GL. Tumors were harvested to prepare paraffin tissue sections for immunofluorescent staining.

### Statistics

Differences in quantitative variables were analyzed by the Mann–Whitney *U* test when comparing two groups and by the Kruskal–Wallis with Dunn’s post hoc test when comparing more than two groups. All analyses were performed using GraphPad Prism software. Survival curves were generated according to the Kaplan**–**Meier method, and statistical analysis was performed using the log-rank test. The association between HMGB1 expression and clinicopathological characteristics was tested by Pearson’s Chi-Square test. *p* value < 0.05 was considered statistically significant.

## Supplementary Information

Below is the link to the electronic supplementary material.

Supplementary Table 1 Correlation between HMGB1 expression and clinicopathological features in primary ESCC. Supplementary Table 2 Correlation between combined prognostic factors of HMGB1 expression (intratumoral) and proliferating Ki67+CD20+ B-cell expression (peritumoral) with clinicopathologic features in primary TMA samples of ESCC. Patients were divided into four subgroups among 74 cases which showed positive peritumoral CD20+ cells. Subgroups were divided according to the expression in two compartments: intratumoral HMGB1 and peritumoral %CD20+Ki67+/total CD20+, Subgroup 1: HMGB1 and CD20+Ki67+ counts were both low (n = 27). Subgroup 2: CD20+Ki67+ count was high but low HMGB1 (n = 34). Subgroup 3: CD20+Ki67+ count was low but high HMGB1 (n = 33). Subgroup 4: HMGB1 and CD20+Ki67+ counts were both high (n = 31). Supplementary Table 3 Primer sequences used for real‐time quantitative polymerase chain reaction analyses (PDF 166 KB)

Supplementary Figure 1 a Proliferating B cells (%CD20+Ki67+ cells/total CD20+ cells) in 74 paired tumor and normal tissues of ESCC. Line colors indicate the directionality of the change (red indicates a decreased abundance in the tumor relative to the normal, while black indicates an increased abundance in the tumor relative to the normal). b Mononuclear cells freshly isolated from four paired ESCC tissues and stained with antibody against CD3 (pan–T cell biomarker) and analyzed by flow cytometry. Stacked bar plot indicates the high fractions of CD3– than CD3+ immune content in both normal and tumor tissues. c Higher fractions of CD3– than CD3+ immune content were observed in tissues of RNA sequencing data from three paired ESCC tumor and non-tumor tissues. d Columns showing the proportion of 22 types of infiltrating immune cells in three pairs of ESCC tumor tissues. The columns represent each patient sample, and the proportions of the immune cells are shown in different colors using the CIBERSORT algorithm with permutations set at 100 and with LM22, which included 22 immune cell types as gene signature reference. e ESCC proliferating tumor-infiltrating B cells (TIL-Bs). Mononuclear cells freshly isolated from three ESCC tissues were analyzed by FACS to quantify the proliferating B cells based on DAPI (live cells marker), CD45 (lymphocyte marker), CD20 (B-cell marker), and Ki67 (proliferation marker). Viable cells were assessed for % proliferating TIL-Bs in B-cell compartment. f Quantification of proliferating TIL-Bs in different subsets based on Ki67 expression: naïve B cells (IgD+ CD27–), memory B cells (IgD–CD27+), unswitched memory (IgD+CD27+), plasmablasts and/or plasma cells, and (CD38+CD27+). The bar plot shows the percentage of proliferating cells in different subsets of TIL-Bs. g Quantification of the density of total B cells and h total proliferating B cells as counts/mm2 in 74 paired ESCC tumor (orange) and normal (blue) whole tissues mass. Supplementary Figure 2 a A chart detailing cases used in tissue microarray (TMA) staining and the number of patients selected for respective analysis were presented in different colors. Two TMAs (TMA-1 and TMA-2) were used in the study with a total of 140 cases. 15 cases that were absent from CD20 immunostaining were excluded throughout the study. The value in parentheses represents the number of tumor/adjacent normal pairs from ESCC patients. b Differences in the intensity (AU) data of HMGB1 expression from 89 cores of TMA by comparing pairs tumor and normal tissues of TMA. c HMGB1 expression was upregulated in tumor tissues in RNA-seq data of three paired ESCC tumor and non-tumor tissues. d qRT-PCR analysis on HMGB1 mRNA expression in non-paired tumor and normal tissue in 38 patients with ESCCs. e Heatmap association for intratumoral HMGB1 protein expression, peritumoral B cells (CD20), and peritumoral proliferating B cells (CD20Ki67). Supplementary Figure 3 a Representative region of interest (ROI) from three independent experiments. B cells were freshly isolated from PBMC and incubated with recombinant HMGB1 (rHMGB1) for 6-h cytospin, followed by staining with a fluorescently labeled antibody specific for HMGB1 (green) and CD20 (red) and examined by confocal microscopy. Nuclei stained with DAPI (blue). Scale bar = 50 μm. Mander’s overlap coefficient (MOC) ± SEM for the surface overlap was calculated by analyzing 12 cellular images (four images per sample, experiments performed in triplicate). b B cells were stimulated with 100 ng/mL recombinant IL-4 and 5 µg/mL IgM in the presence of rHMGB1(10 ng/mL) for 72 h and analyzed for the expression of Ki67. c Expression of HMGB1 was detected in seven ESCC cell lines and one immortalized esophageal epithelial cell line (NE1) by western blot. d Cells were transfected with pcDNA (empty vector) or pcDNA-HMGB1. Overexpression of HMGB1 in stable transfected tumor cells (EC18H and K510H) was confirmed by western blot (top panel) and PCR analysis (bottom panel). e The relative expression of HMGB1 in overexpressed tumor cells (EC18H and K510H) and vector control (EC18pc and K510pc) was detected by qRT-PCR. f Representative plots showing the progressive gating strategy for analysis of CD20+ B cells in CD20-positive selected magnetic bead-enriched lymphocytes. Purities of 98% enriched CD20+ cells were achieved. Supplementary Figure 4 a FACS gating on CD20+ B-cell populations in CFSE-labeled PBMC co-cultured with HMGB1 (EC18H and K510H) /empty vector-transfected tumor cells (EC18pc and K510pc) for 6 days. b CFSE-labeled B cells were pre-stimulated with 100 ng/mL recombinant IL-4 and 5 µg/mL IgM, washed, and co-cultured with the indicated tumor cells for 6 days. Histograms show the degree of CFSE dilution (division slicing), indicative of B-cell proliferation. Data shown are representative of three repeats. c Representative density plots showing co-expression of CD20+Ki67+ on B cells upon co-culture for 24 h. d Representative double staining of B cells (CD20; pink) and HMGB1 (brown) in ESCC tumor tissues. e Optimization of the concentration of recombinant HMGB1 for chemotaxis. f Optimization of the number of B cells for chemotaxis assay. Chemotactic index is the ratio of the number of cells in the bottom chamber in the test case (recombinant HMGB1) to the number of cells in the absence of the drug (medium only). (*P < 0.05; n = 3). Supplementary Figure 5 a Example showing B cells stained with CFSE and co-cultured with HMGB1-overexpressing/vector tumor cells for 6 days. B cells with high (hi) and low (lo) CFSE signal (slow and fast proliferation, respectively) were sorted by FACS for RNA isolation. B cells exhibited with CFSElo profile (black bar) expressed higher VEGF as compared to vector control (EC18pc, K510pc) (#P < 0.05) and CFSEhi subsets (gray bar) (**P < 0.01). b Quantification of VEGF and Ki67 expression by FACS in B cells upon co-culturing with HMGB1-expressing tumor cells or vector control. c Bar chart of the relative percentage of proliferating VEGF-producing B cells of 51 ESCC patients. No CD20+VEGF+ B cells are found in 8 patients: #3, 9, 12, 25, 34, 35, 44, and 55. CD20+VEGF+ Ki67+ B cells are indicated by teal and CD20+VEGF+ Ki67- B cells are indicated by dark green. d Spatial association between CD20+ B cells and CD31+ blood vessels were identified in consecutive 10 µm steps (distance classes) within 200 µm. Supplementary Figure 6 a HUVEC spheroids were monitored and cultured up to 72 h with CM under three different conditions: B cells co-cultured with vector cells and HMGB1-overexpressing tumor cells in the presence or absence of GL. Representative images of phase-contrast micrographs of HUVEC spheroids were shown. Images of a spheroid sprouting area from EC18H/B at 72 h (top right) with magnifications of highlighted regions (bottom right) were shown. Scale bar represents 100 μm. b The top panel shows images of the sprouts after threshold settings for each segment and corresponding resulting binary images. The areas covered by spheroids are outlined in yellow. The lower panel shows images “skeletonize” function of Fiji (ImageJ). c Quantification of branching length per spheroid and sprout length. d Movement of HUVECs in a chemotactic gradient of co-culture with HMGB1-expressing tumor cells and B cells is reduced by the presence of GL, as shown by a representative cell tracking analysis. Supplementary Figure 7 a Incubation of HUVECs with CM collected from co-cultures (EC18pc/B and K510pc/B vs. EC18H/B and K510H/B) in the presence (+) or absence (−) of anti-HMGB1 monoclonal antibody (αHMGB1, 10 µg/mL). HUVEC migration and tube formation were counted by ImageJ. Scale bar: 200 µm. b Cell supernatants collected from tumor cells treated in the presence (+) or absence (−) of αHMGB1 were subjected to western blotting for detecting HMGB1 soluble protein. Coomassie brilliant blue-stained gels were used to confirm equal loading. c The levels of phosphorylated ERK, p38, and VEGF were downregulated in B cells upon addition of GL in the co-culture with HMGB1-overexpressing cells. d Cell supernatants collected from tumor cells treated in the presence (+) or absence (−) of anti-VEGF monoclonal antibody (αVEGF, 10 µg/mL) were subjected to western blotting for detecting VEGF soluble protein. Coomassie brilliant blue-stained gels were used to confirm equal loading. e Incubation of HUVEC with CM collected from B cells pre-treated with IL4/IgM (+) or left untreated (−) in the presence (+) or absence (−) of anti-VEGF monoclonal antibody. HUVEC migration and tube formation were counted by ImageJ. Scale bar: 200 µm. f The dissociated tumor cell population was sorted by FACS based on Epcam (epithelial cell marker), CD45 (immune cell marker), and CD20 (B-cell marker) and performed qRT-PCR on sorted B cells. Supplementary Figure 8 a qRT-PCR analysis on B cells collected from co-culture with HMGB1-overexpressing (EC18H/K510H) or vector cells. b Representative flow cytometric analysis of cell surface molecules on CD19 and CD27/CD38 on B cells co-cultured with HMGB1-overexpressing cells or vector controls. c Supernatant was collected from co-cultures to evaluate the level of IgG and IgM by ELISA (*P < 0.05, **P < 0.01). Results are presented as mean ± SEM of at least three experiments. d Representative image of cancer tissue showing few CD138+ plasma cells aggregates in cancer stroma. Scale bar: 100µm (PDF 2247 KB)
