# Supplementary material for: Peritumoral B cells drive proangiogenic responses in HMGB1-enriched esophageal squamous cell carcinoma
Source: Angiogenesis. 2021 Oct 6;25(2):181–203. doi: 10.1007/s10456-021-09819-0 (PMC8494172; doi:10.1007/s10456-021-09819-0)
Supplement: Supplementary file 1 — Supplementary Table 1 Correlation between HMGB1 expression and clinicopathological features in primary ESCC. Supplementary Table 2 Correlation between combined prognostic factors of HMGB1 expression (intratumoral) and proliferating Ki67+CD20+ B-cell expression (peritumoral) with clinicopathologic features in primary TMA samples of ESCC. Patients were divided into four subgroups among 74 cases which showed positive peritumoral CD20+ cells. Subgroups were divided according to the expression in two compartments: intratumoral HMGB1 and peritumoral %CD20+Ki67+/total CD20+, Subgroup 1: HMGB1 and CD20+Ki67+ counts were both low (n = 27). Subgroup 2: CD20+Ki67+ count was high but low HMGB1 (n = 34). Subgroup 3: CD20+Ki67+ count was low but high HMGB1 (n = 33). Subgroup 4: HMGB1 and CD20+Ki67+ counts were both high (n = 31). Supplementary Table 3 Primer sequences used for real‐time quantitative polymerase chain reaction analyses (PDF 166 KB) [file 10456_2021_9819_MOESM1_ESM.pdf]

Supplementary Table 1

	Total (n = 118)	HMGB1 expression		P value
		Low expression (n = 59)	High expression (n =59)	
**Gender**				0.708
Male	70	34	36	
Female	48	25	23	
**Age**				0.853
<60	67	34	33	
≥60	51	25	26	
**Differentiation**				0.067
Well	12	5	7	
Modest	74	43	31	
Poor	32	11	21	
**Stage**				0.527
0	1	1	0	
I	3	1	2	
IIA	77	41	36	
IIB	12	4	8	
III	24	11	13	
IVB	1	1	0	
**T stage**				0.587
T0	1	1	0	
T1	3	1	2	
T2	44	20	24	
T3	69	36	33	
T4	1	1	0	
**N stage**				0.035
Nx	6	6	0	
N0	75	37	38	
N1	37	16	21	

Supplementary Table 2

		Subgroup 1	Subgroup 2	Subgroup 3	Subgroup 4	
	Total (n = 125)	Both 1ow (n = 27)	Low HMGB1/ High CD20Ki67 (n = 34)	High HMGB1/ Low CD20Ki67 (n = 33)	Both high (n = 31)	P value
**Gender**						0.656
Male	78	15	22	23	18	
Female	47	12	12	10	13	
**Age**						0.586
<60	64	11	17	18	18	
≥60	61	16	17	15	13	
**Differentiation**						0.093
Well	14	1	2	4	7	
Modest	78	19	19	24	16	
Poor	33	7	13	5	8	
**Stage**						0.258
0	1	1	0	0	0	
I	3	1	2	0	0	
IIA	63	11	20	19	13	
IIB	12	0	4	3	5	
III	42	12	6	11	13	
IVA	2	1	1	0	0	
IVB	2	1	1	0	0	
**T stage**						0.205
T0	1	1	0	0	0	
T1	3	1	2	0	0	
T2	38	5	15	9	9	
T3	82	20	16	24	22	
T4	1	0	1	0	0	
**N stage**						0.055
Nx	5	1	0	4	0	
N0	62	12	22	15	13	
N1	58	14	12	14	18	

Supplementary Table 3

qRT-PCR primer	
**HUMAN**	
**VEGFA-F**	5'ATCTGCATGGTGATGTTGGA'3
**VEGFA-R**	5'GGGCAGAATCATCACGAAGT'3
**EDN1-F**	5'AGAGTGTGTCTACTTCTGCCA'3
**EDN1-R**	5'CTTCCAAGTCCATACGGAACAA'3
**VEGFR2-F**	5'GTGATCGGAAATGACACTGGAG'3
**VEGFR2-R**	5'CATGTTGGTCACTAACAGAAGCA'3
**CD31-F**	5'ACCGTGACGGAATCCTTCTCT'3
**CD31-R**	5'GCTGGACTCCACTTTGCAC'3
**PAX5-F**	5'ACTTGCTCATCAAGGTGTCAG'3
**PAX5-R**	5'TCCTCCAATTACCCCAGGCTT'3
**IRF4-F**	5'GCTGATCGACCAGATCGACAG'3
**IRF4-R**	5'CGGTTGTAGTCCTGCTTGC'3
**BCL2-F**	5'GGTGGGGTCATGTGTGTGG'3
**BCL-R**	5'CGGTTCAGGTACTCAGTCATCC'3
**BACH2-F**	5'GCACATTCCGGGAAGATAACTC'3
**BACH2-R**	5'GGGCGGCTCACTTTTAATCTG'3
**IL8-F**	5'ACCGGAAGGAACCATCTCAC'3
**IL8-R**	5'AAACTGCACCTTCACACAGAG'3
**HMGB1-F**	5'GCGGACAAGGCCCGTTA'3
**HMGB1-R**	5'AGAGGAAGAAGGCCGAAGGAA'3
**beta-actin-F**	5'AGCACAGAGCCTCGCCTTT'3
**beta-actin-R**	5'ATCATCATCCATGGTGAGCTGG'3
**CXCR5-F**	5'CACGTTGCACCTTCTCCCAA'3
**CXCR5-R**	5'GGAATCCCGCCACATGGTAG'3
**CCR7-F**	5'GATACCTACCTGCTCAACCTG'3
**CCR7-R**	5'AAGTGGACACCGAAGACCC'3
**MICE**	
**vegfr1-F**	5’TGGACCCAGATGAAGTTCCC’3
**vegfr1-R**	5’GCGATTTGCCTAGTTTCAGTCT’3
**vegfr2-F**	5’AGCACTGGTCCTATGGGTTG’3
**vegfr2-R**	5’GGTTCTGCCATTTGATCCA’3
**fgfr1-F**	5’ATACGTGTTGGCGGGTAACTC’3
**fgfr2-R**	5’TGGAGTGGTCATCGCCACCTTAAT’3
**gapdh-1**	5’AGGTCGGTGTGAACGGATTTG’3
**gapdh-2**	5’TGTAGACCATGTAGTTGAGGTCA’3
**Transfection and cloning of HMGB1 expression constructs**	
**HMGB1-CLO-F-NheI**	5'CGGCTAGC TTCGGTGCACGAAAAATAACTAA'3
**HMGB1-CLO-R-XhoI**	5'CGCTCGAG AGTGAGTTGTGTACAGGGGG'3
