# Supplementary material for: Peritumoral B cells drive proangiogenic responses in HMGB1-enriched esophageal squamous cell carcinoma
Source: Angiogenesis. 2021 Oct 6;25(2):181–203. doi: 10.1007/s10456-021-09819-0 (PMC8494172; doi:10.1007/s10456-021-09819-0)

Supplementary Figure 1

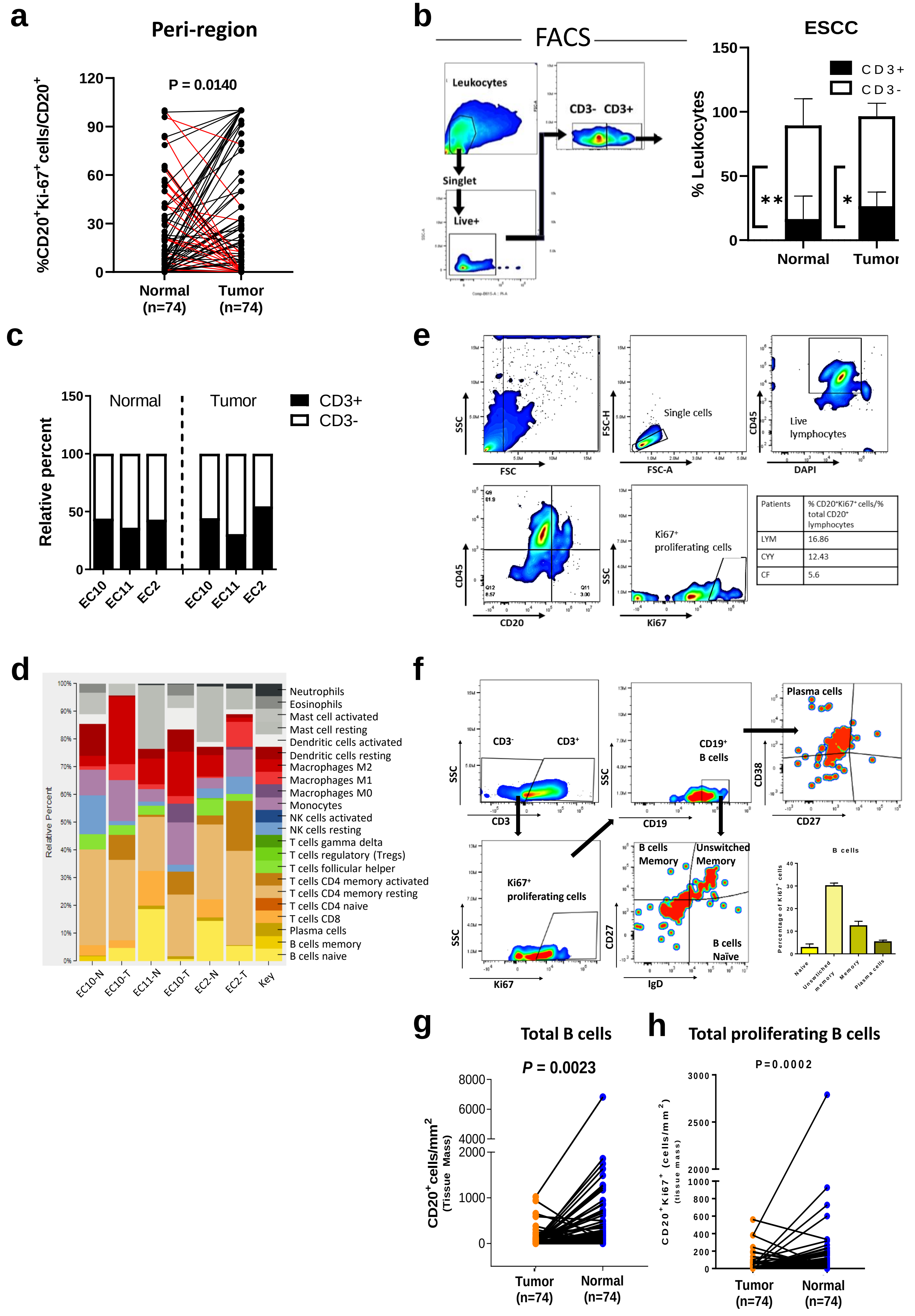

Patients	% CD20⁺Ki67⁺ cells/% total CD20⁺ lymphocytes
LYM	16.86
CYY	12.43
CF	5.6

Supplementary Figure 2

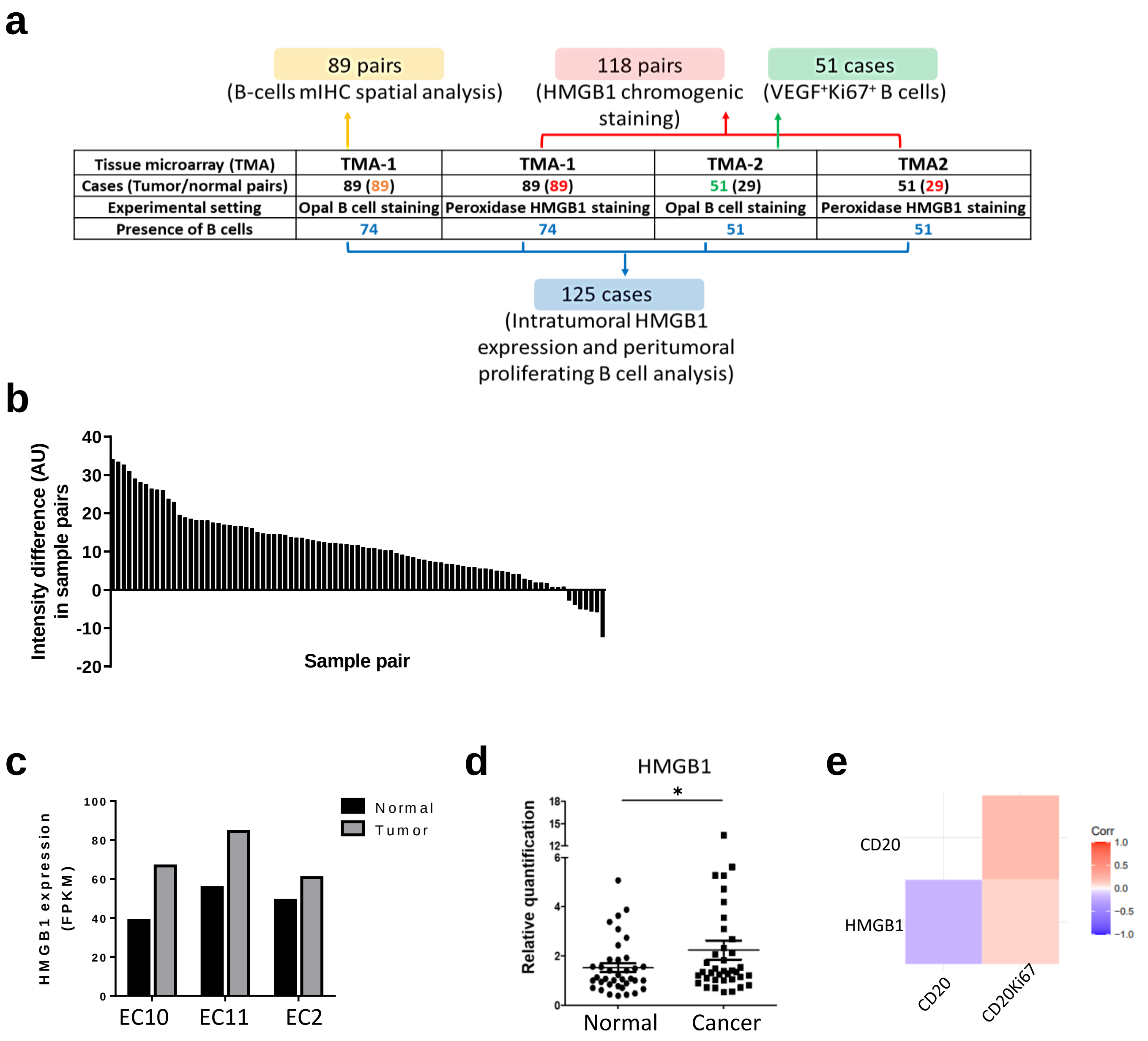

Supplementary Figure 3

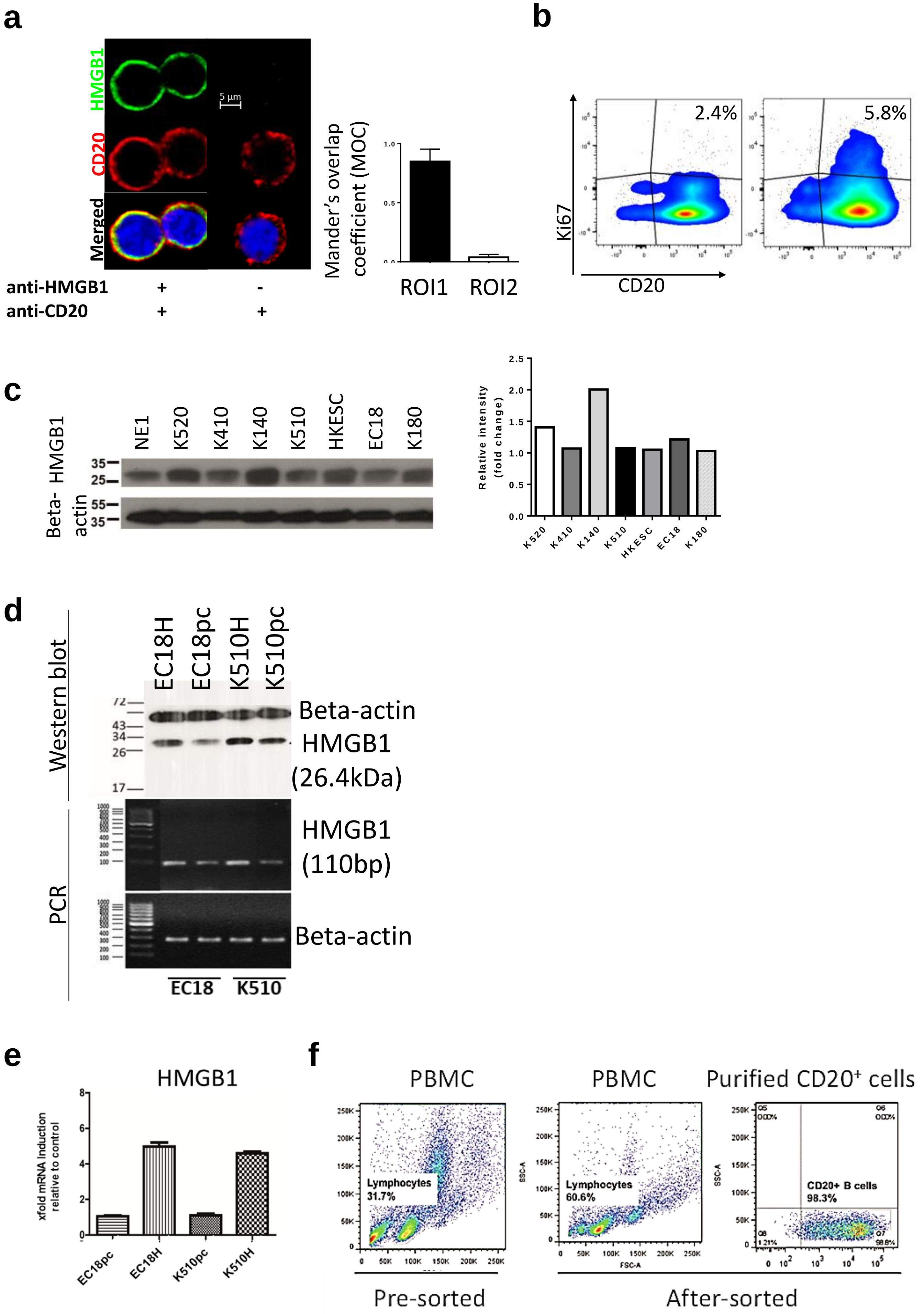

Supplementary Figure 4

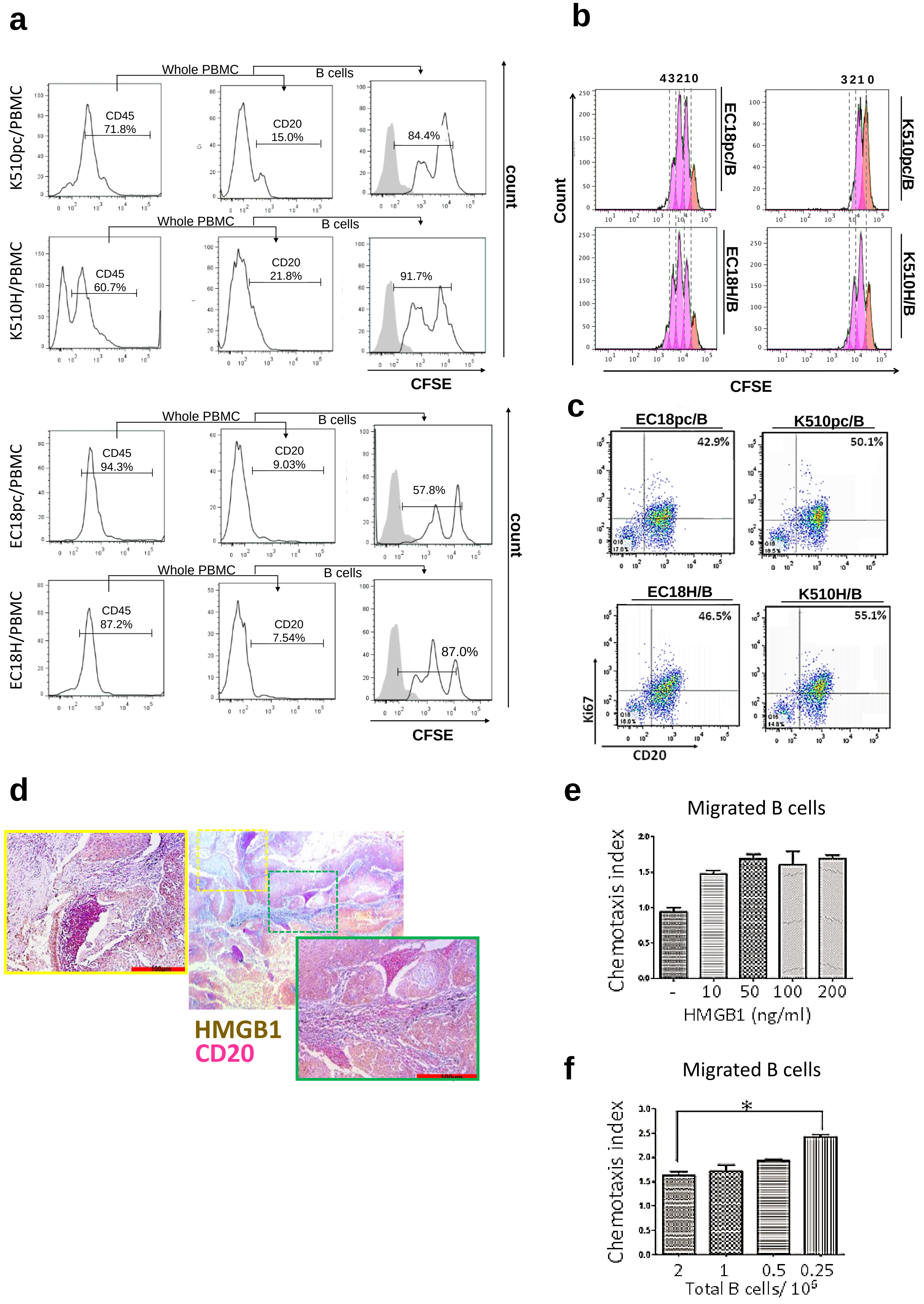

Supplementary Figure 5

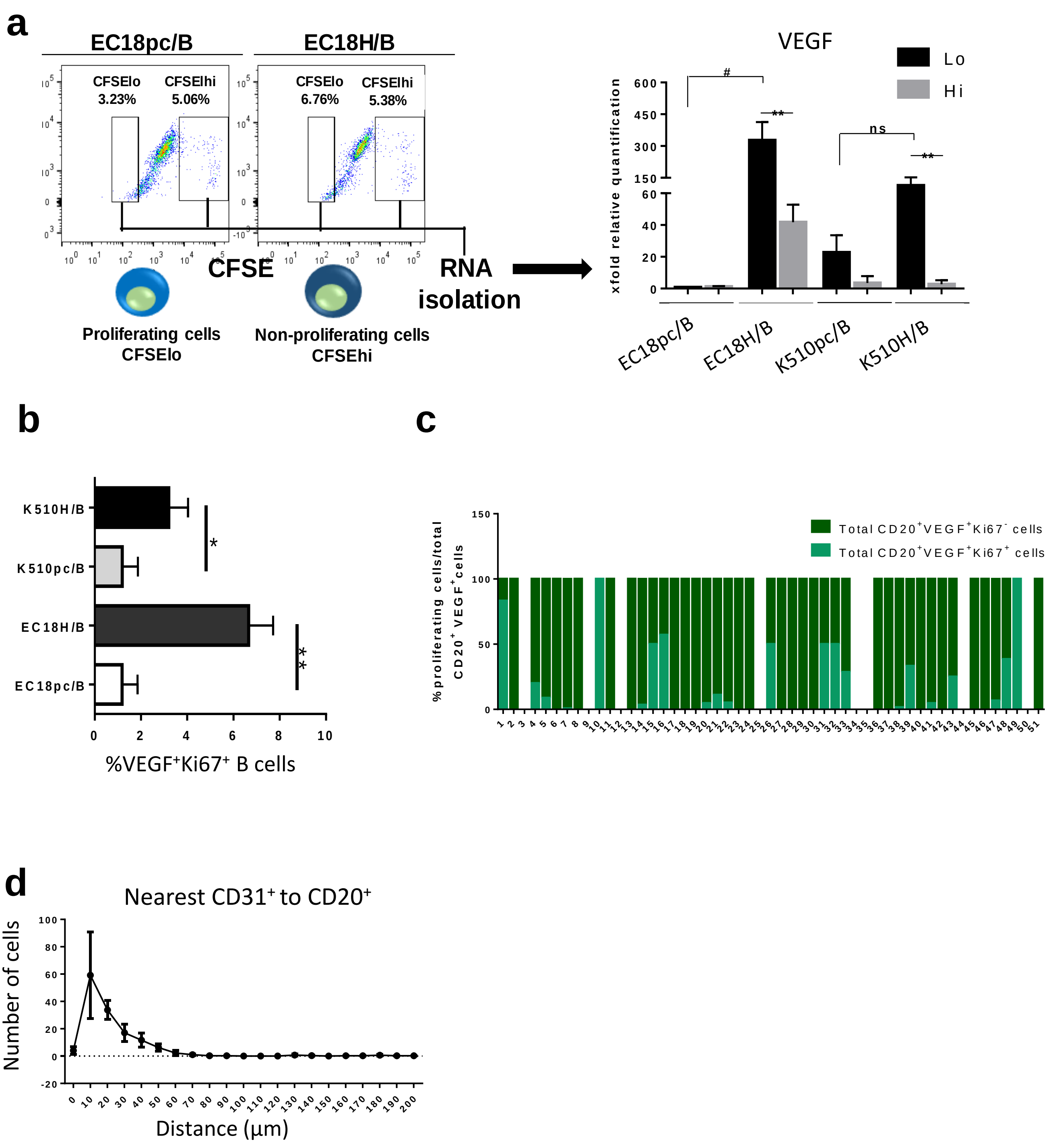

Supplementary Figure 6

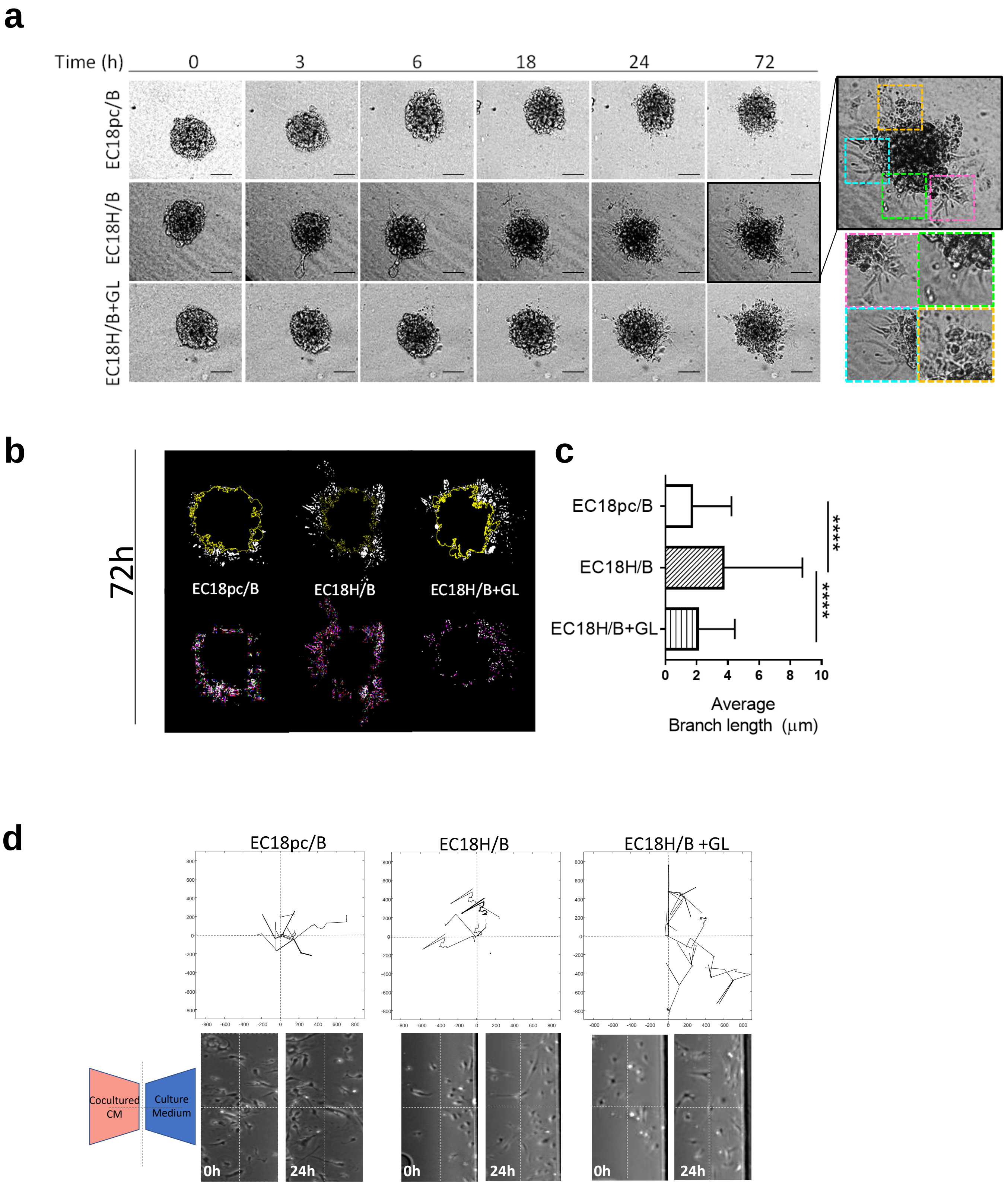

Supplementary Figure 7

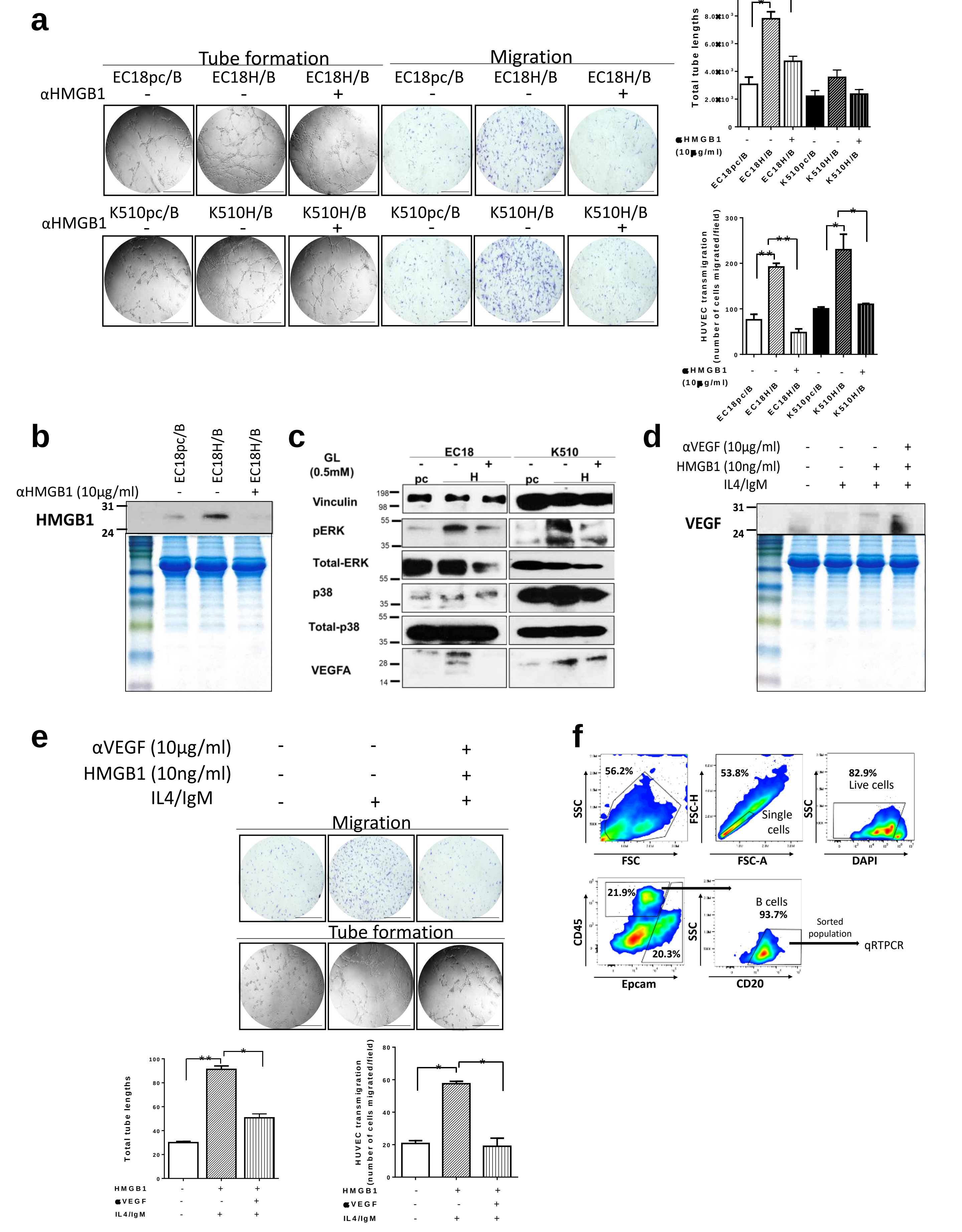

Supplementary Figure 8

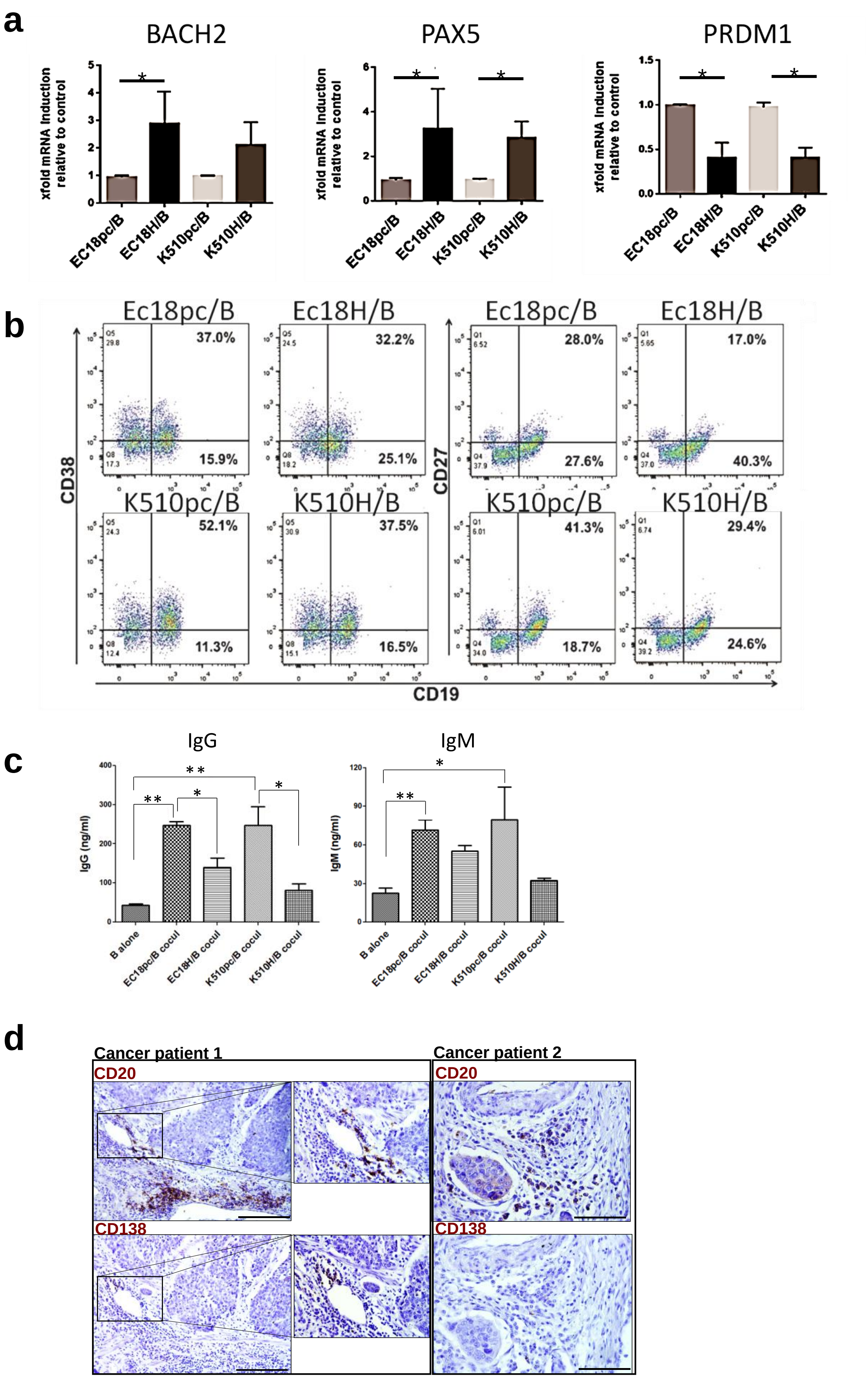

Supplement: Supplementary file 2 — Supplementary Figure 1 a Proliferating B cells (%CD20+Ki67+ cells/total CD20+ cells) in 74 paired tumor and normal tissues of ESCC. Line colors indicate the directionality of the change (red indicates a decreased abundance in the tumor relative to the normal, while black indicates an increased abundance in the tumor relative to the normal). b Mononuclear cells freshly isolated from four paired ESCC tissues and stained with antibody against CD3 (pan–T cell biomarker) and analyzed by flow cytometry. Stacked bar plot indicates the high fractions of CD3– than CD3+ immune content in both normal and tumor tissues. c Higher fractions of CD3– than CD3+ immune content were observed in tissues of RNA sequencing data from three paired ESCC tumor and non-tumor tissues. d Columns showing the proportion of 22 types of infiltrating immune cells in three pairs of ESCC tumor tissues. The columns represent each patient sample, and the proportions of the immune cells are shown in different colors using the CIBERSORT algorithm with permutations set at 100 and with LM22, which included 22 immune cell types as gene signature reference. e ESCC proliferating tumor-infiltrating B cells (TIL-Bs). Mononuclear cells freshly isolated from three ESCC tissues were analyzed by FACS to quantify the proliferating B cells based on DAPI (live cells marker), CD45 (lymphocyte marker), CD20 (B-cell marker), and Ki67 (proliferation marker). Viable cells were assessed for % proliferating TIL-Bs in B-cell compartment. f Quantification of proliferating TIL-Bs in different subsets based on Ki67 expression: naïve B cells (IgD+ CD27–), memory B cells (IgD–CD27+), unswitched memory (IgD+CD27+), plasmablasts and/or plasma cells, and (CD38+CD27+). The bar plot shows the percentage of proliferating cells in different subsets of TIL-Bs. g Quantification of the density of total B cells and h total proliferating B cells as counts/mm2 in 74 paired ESCC tumor (orange) and normal (blue) whole tissues mass. Supplementary [file 10456_2021_9819_MOESM2_ESM.pdf]
